# Inhibiting TNAP Attenuates the Aortic Valve Calcification and Reduces Oxidative Stress

**DOI:** 10.3390/cells15171543

**Published:** 2026-08-26

**Authors:** Hyeshin Kwon, Hak Su Kim, Minjeong Kwon, Soyoung Jo, Giwon Hwang, Sae-Kwang Ku, Ilwhea Ku, Yong Hwa Jo

**Affiliations:** 1 Research and Development, REDNVIA Co., Ltd., Seoul 08511, Republic of Korea; 2 Veterans Medical Research Institute, Veterans Health Service Center, Seoul 05368, Republic of Korea; 3 College of Korean Medicine, Daegu Hanny University, Gyeongsan 38610, Republic of Korea

**Keywords:** tissue-nonspecific alkaline phosphatase, TNAP, alkaline phosphatase tissue non-specific, ALPL, calcific aortic valve disease, CAVD, valve calcification, oxidative stress, antioxidants

## Abstract

**Highlights:**

**What are the main findings?**

- Novel TNAP inhibition significantly attenuates aortic valve calcification by lowering elevated TNAP levels and reducing hydroxyapatite crystallization in a vitamin D3-induced CAVD mouse model.
- TNAP inhibitors restore the valvular antioxidant defense system (GSH, SOD, CAT) while markedly suppressing lipid peroxidation (MDA, 4-HNE, MPO), pro-inflammatory cytokines (IL-1β, TNF-α), and apoptotic pathways (cleaved Cas-3, cleaved PARP).

**What are the implications of the main findings?**

- Direct targeting of TNAP offers a viable, disease-modifying pharmacological strategy for advanced-stage CAVD, addressing the critical clinical gap where surgical or transcatheter valve replacement is currently the only option.
- TNAP inhibition represents a promising therapeutic approach that successfully halts pathological cardiovascular remodeling (calcific, fibrotic, and osteogenic pathways) without compromising normal skeletal bone homeostasis in short-term models.

**Abstract:**

Calcific aortic valve disease (CAVD) is a progressive condition driven by oxidative stress, chronic inflammation, and the osteogenic reprogramming of valvular interstitial cells, leading to hydroxyapatite crystallization. Because tissue non-specific alkaline phosphatase (TNAP) drives phosphate-mediated mineralization, this study evaluated the efficacy of novel TNAP inhibitors as a disease-modifying strategy. Using a vitamin D3-induced CAVD mouse model presenting human-like annular thickening and valvular calcification, we analyzed the therapeutic impact of these inhibitors. TNAP expression was highly elevated in diseased valves; however, inhibitor treatment significantly lowered TNAP levels and attenuated valvular calcification. Notably, the inhibitors suppressed lipid peroxidation and restored antioxidant capacity, significantly regulating GSH, SOD, and CAT levels while decreasing lipid oxidation markers (MDA, 4-HNE, MPO). Furthermore, pro-inflammatory cytokines (IL-1β, TNF-α) and apoptotic markers (cleaved Cas-3, cleaved PARP) were markedly decreased, accompanied by diminished fibrotic and osteogenic remodeling, while maintaining normal bone homeostasis. In conclusion, TNAP inhibition effectively reduces pathological valve calcification by suppressing interconnected oxidative, inflammatory, fibrotic, and osteogenic pathways, positioning it as a promising and safe therapeutic approach for CAVD.

## 1. Introduction

Calcified aortic valve disease (CAVD) is the most common of valvular heart disease, characterized by progressive leaflet thickening, fibrosis, and calcification. This pathological process can ultimately lead to adverse cardiovascular outcomes, including severe heart failure and sudden cardiac death [1,2]. Globally, the burden of CAVD has increased substantially over the past three decades, with the number of newly diagnosed cases rising 3.51-fold from 1990 to 2019, reaching approximately 589,638 cases in 2019. Furthermore, the incidence rate exhibited a 6.54-fold increase among populations in the middle sociodemographic index (SDI) quintile during this period [3]. Despite its increasing prevalence in the aging population, there are currently no pharmacological therapies available to delay or reverse valvular calcification, and surgical or transcatheter valve replacement remains the only effective treatment option.

As the most dynamically active tissue in the heart, the aortic valve is constantly exposed to diverse mechanical and biochemical stresses. Shear stress induces an imbalance between oxidative stress and endogenous antioxidant defense mechanisms, orchestrating the transition of valvular interstitial cells (VICs) to osteoblast-like cells. In the pathological microenvironment of CAVD, the infiltration of inflammatory cells leads to an elevated expression of myeloperoxidase (MPO), which accelerates the production of reactive oxygen species (ROS) and reactive nitrogen species (RNS) [4]. This overwhelming oxidative surge progressively dampens the tissue’s primary antioxidant defenses and inactivates the cytoprotective nuclear factor erythroid 2-related factor 2 (Nrf2) signaling pathway [5]. Consequently, chronic oxidative stress triggers downstream pro-inflammatory and pro-osteogenic cascades, notably through the activation of nuclear factor kappa B (NF-κB), which fuels extensive molecular damage and lipid peroxidation within the valvular matrix [4]. These oxidative and nitrative modifications ultimately serve as potent intracellular stimuli, driving the phenotypic reprogramming and osteogenic differentiation of VICs.

To identify potential therapeutic targets for CAVD, a range of molecular targets have been proposed, primarily focusing on fibrosis-, oxidative stress–, and inflammation-related pathways, including basic fibroblast growth factor (bFGF), ENPP-2 (autotaxin), NADPH oxidase 2, Toll-like receptor 3 (TLR3), and interleukin-21 (IL-21) [6,7,8,9,10]. Other researchers have focused on metabolic disorders, such as diabetes, and have investigated the application of anti-diabetic agents, including metformin and gliptins [11,12]. Most therapeutic approaches have focused on early-stage CAVD, whereas relatively few strategies have been developed to directly suppress calcification, which represents the advanced stage of disease progression.

A key biochemical driver of pathological calcification is tissue non-specific alkaline phosphatase (TNAP), which is a membrane-bound ectoenzyme that hydrolyzes pyrophosphate (PPi), an endogenous inhibitor of biomineralization [13]. By reducing PPi and increasing inorganic phosphate (Pi), TNAP directly promotes mineral nucleation and subsequent hydroxyapatite growth [14,15,16]. Recently, TNAP has also been shown to contribute to passage-dependent calcification in VICs, where its activity correlates with mineralization under osteogenic conditions [17]. In the WHC-eTNAP mouse model, ectopic calcification was accelerated, whereas treatment with prototypical TNAP inhibitor SBI-425 significantly reduced coronary calcium burden and improved ejection fraction [18]. Notably, local activation of TNAP within arteries appears to promote vascular calcification as TNAP overexpression in vascular smooth muscle cells (VSMCs) and endothelial cells has been shown to induce calcific remodeling [19]. Consistent with this, endothelial-specific TNAP overexpression mice resulted in generalized arterial calcification and left ventricular hypertrophy with preserved ejection fraction through deposition of Runx2, osteocalcin, and osteopontin [20].

In this study, we developed a novel TNAP inhibitor and evaluated its therapeutic efficacy in an experimental model of CAVD. Using in vivo approaches, we investigated the effects of TNAP inhibition on valvular calcification, aiming to establish TNAP as a viable pharmacological target for the treatment of advanced-stage CAVD.

## 2. Materials and Methods

### 2.1. Cell Culture

VICs were isolated from patients with CAVD undergoing aortic valve replacement. Human aortic valve tissues were washed with phosphate-buffered saline (PBS) and incubated in Dulbecco’s modified Eagle medium (DMEM) containing a collagenase cocktail for 1 h at 37 °C with gentle stirring to remove valvular endothelial cells (VECs). The tissues were then minced with a blade and further incubated in DMEM containing collagenase cocktail and dispase overnight at 37 °C with continuous stirring. The digested cell suspension was collected, sequentially filtered through 70 μm and 40 μm cell strainers, and centrifuged at 12,000 rpm for 5 min. After removal of the supernatant, the cell pellet was resuspended and cultured. Patient-derived VICs were maintained in DMEM (Gibco, Grand Island, NY, USA) supplemented with 10% FBS (Gibco, Grand Island, NY, USA), 100 U/mL penicillin, and 100 μg/mL streptomycin (Gibco, Grand Island, NY, USA) at 37 °C in a humidified atmosphere containing 5% CO_2_.

The study protocol for the collection of human samples was approved by the Institutional Review Board (IRB) of Asan Medical Center (Seoul, Republic of Korea) (IRB No. 2017-0556), and written informed consent was obtained from all patients.

### 2.2. TNAP Inhibitors and IC_50_ Calculation

The established TNAP inhibitors MLS0038949, SBI-425, and DS-1211 were used in this study as reference compounds [21,22,23]. RNV-1002 was newly synthesized in-house by our group. The half-maximal inhibitory concentration (IC_50_) values for the panel of TNAP inhibitors developed in this study are provided in Supplementary Table S1. Compound #178 was designated as RNV-1002 and selected for further biological evaluation.

TNAP inhibitory activity was determined using a colorimetric alkaline phosphatase assay kit (Abcam, Cambridge, UK) and recombinant human ALPL (BioLegend, San Diego, CA, USA). Recombinant ALPL (2 ng/well) was incubated with test compounds for 10 min at room temperature in the dark. The reaction was initiated by adding p-nitrophenyl phosphate (pNPP) substrate (final concentration, 0.8 mM) and incubated for 120 min at room temperature in the dark. Absorbance was measured at 405 nm using a microplate reader. IC_50_ values were calculated by nonlinear regression using GraphPad Prism software (ver.10).

### 2.3. Osteogenesis and Measurement

VICs were isolated from three individual patients (*N* = 3). To induce osteogenic differentiation, VICs were cultured in osteogenic induction medium consisting of DMEM supplemented with 10% FBS, 0.1 μM dexamethasone, 50 μM ascorbic acid, and 10 mM β-glycerophosphate. The medium was replaced every 2–3 days, and cells were maintained for 21–28 days at 37 °C in a humidified atmosphere containing 5% CO_2_.

For osteogenic differentiation of SaOS-2 cells, cells were cultured in the same osteogenic medium for 7 days under identical conditions.

All assays were performed in technical duplicates (*n* = 2) for each biological donor. To avoid pseudo-replication, values from technical duplicates were averaged to generate a single representative value per biological replicate prior to statistical testing (*n* = 3). To verify reproducibility, all experiments were independently repeated by a second investigator following the identical protocol.

### 2.4. Alizarin Staining

For Alizarin Red S staining, cells were fixed with 4% paraformaldehyde for 15 min and then stained with Alizarin Red S solution (1% Alizarin Red S, pH 4.2) for 30 min at room temperature. After staining, the cells were washed with distilled water to remove excess dye. For quantitative analysis, the stained cells were destained with 10% cetylpyridinium chloride (CPC) solution for 1 h with gentle rotation at room temperature. The extracted solution (100 μM) was transferred to a 96-well plate, and absorbance was measured at 560 nm using a microplate reader.

### 2.5. Cell Viability

Cell viability was measured using the EZ-Cytox Cell Viability Assay kit (DoGenBio, Seoul, Republic of Korea) according to the manufacturer’s instructions. Briefly, cells were incubated with 10% (*v*/*v*) EZ-Cytox solution for 30 min at room temperature. After incubation, 100 μL of the supernatant was transferred to a 96-well plate, and absorbance was measured at 450 nm using a microplate reader.

### 2.6. Establishment of Vitamin D-Induced CAVD Mice

Male C57BL/6 mice were administered vitamin D_3_ (6.5 × 10^5^ IU/Kg) by subcutaneous injection once daily for 3 consecutive days. Vitamin D_3_ was dissolved in a vehicle consisting of polyethylene glycol 400 (PEG400), 0.5% Tween-80, 5% propylene glycol (PG), and 64.5% saline, and injected at a volume of 10 mL/kg. After the final injection, mice were maintained for an additional 5 days to allow the development of CAVD, after which tissues were collected for further analysis. Animal procedures were performed in accordance with international guidelines for the care and use of laboratory animals and were approved by the Institutional Animal Care and Use Committee of Daegu Haany University (approval numbers DHU 2025-006).

### 2.7. Efficacy and Toxicity Test in Vitamin D-Induced CAVD Mice

In the vitamin D-induced CAVD mouse model, the vehicle and test compounds were orally administered once daily for eight consecutive days. After euthanizing the mice, the blood, heart, and aorta were collected for immunohistochemistry, immunostaining, and qPCR analysis.

For the evaluation of high-dose and long-term toxicity, ICR mice were used and treated orally with 100, 200, or 400 mg/kg of the compound for 28 days. Feed and water were supplied freely access. All animals fasted overnight, about 18 h (with free access to water) before first administration and further for 3 h after first administration, and sacrifice. Clinical signs were monitored at 24-h intervals during the entire experimental period. All animal procedures were performed in accordance with international guidelines for the care and use of laboratory animals and were approved by the Institutional Animal Care and Use Committee of Daegu Haany University (approval numbers DHU 2024-056, and DHU 2025-006).

### 2.8. Quantitative PCR

Collected tissues were minced and transferred to Trizol reagent (Invitrogen, Carlsbad, CA, USA) for RNA extraction. To remove contaminating DNA, samples were treated with recombinant DNase I (Thermo Fisher Scientific Inc., Waltham, MA, USA). RNA was reverse transcribed using the reagent High-Capacity cDNA Reverse Transcription Kit (Thermo Fisher Scientific Inc., Waltham, MA, USA) according to the manufacturer’s instructions. The mRNA levels of RANKL, OPG, BMP2, Nrf2, NF-κB, MGP, and β-actin were measured using SYBR-primer premix. The thermal cycler conditions were as follows: Stage 1, 50 °C for 2 min, 95 °C for 10 min; Stage2, 40 cycles of 95 °C for 15 s, 60 °C for 1 min; Stage 3, 95 °C for 15 s, 60 °C for 15 s, 95 °C for 15 s according to the manufacturer’s protocol. All tests were performed in triplicate. Expression data were normalized to geometric mean of housekeeping gene β-actin to control the variability in expression levels and were analyzed using the 2^−ΔΔCT^ method. All qPCR primer pairs were obtained from OriGene Technologies (Rockville, MD, USA), and the sequences of the PCR oligonucleotide primers are listed in Table 1.

### 2.9. Immunohistochemistry and Immunostaining

Approximately equal regions of individual heart around origin/root site of ascending aorta were cross-sectioned as one part in each sample. All cross-sectioned heart-aortic valve regions were re-fixed in 10% neutral buffered formalin for 24 h. Representative paraffin sections were stained with Hematoxylin & Eosin (H&E) for general histopathology, von Kossa for calcium depositions, or ABC-based immunohistochemical analysis for TNAP (ABclonal, Woburn, MA, USA), ENPP (ABclonal, Woburn, MA, USA), cleaved caspase-3 (Cell signaling technology, Danvers, MA, USA), cleaved PARP (Santa cruz biotechnology, Dallas, TX, USA), MPO (ABclonal, Woburn, MA, USA), iNOS (Santa cruz biotechnology, Dallas, TX, USA), COX-2 (Cayman chemical, Ann Arbor, MI, USA), TNF-α (Santa cruz biotechnology, Dallas, TX, USA), IL-1β (Santa cruz biotechnology, Dallas, TX, USA), NT (Millipore, Burlington, MA, USA), 4-HNE (abcam, Cambridge, UK), TGF-β1 (Santa cruz biotechnology, Dallas, TX, USA), and α-SMA (ABclonal, Woburn, MA, USA). Immunoreactive cells were observed by ABC-based immunohistochemical methods using purified primary antibodies (Table 2) with appropriate ABC and peroxidase substrate kit. Endogenous peroxidase activity was blocked by incubating in methanol and 0.3% H_2_O_2_ for 30 min, and non-specific binding of immunoglobulin was blocked with normal horse serum blocking solution for 1 h in humidity chamber after heating (95~100 °C) based epitope retrievals in 10 mM citrate buffers (pH 6.0). Primary antibodies were incubated overnight at 4 °C in humidity chamber, and then incubated with biotinylated universal secondary antibody and ABC reagents for 1 h at room temperature in humidity chamber. Finally, sections were reacted with peroxidase substrate kit for 3 min at room temperature. All sections were rinsed in 0.01 M phosphate buffered saline (PBS) three times, between each step. To evaluate histopathological changes associated with CAVD and ectopic calcification, aortic valve thickness and inflammatory cell infiltration were quantified in H&E-stained sections, and the calcified area was measured in von Kossa (VK)-stained sections using a computer-assisted image analysis system.

### 2.10. Osteoclastogenesis

RAW264.7 cells (ATCC, Manassas, VA, USA) were maintained in DMEM containing 10% FBS (Gibco, Grand Island, NY, USA) at 37 °C in a humidified atmosphere with 5% CO_2_. To differentiate the cells into osteoclast-like cell, RAW264.7 cells were seeded at density of 3 × 10^3^ cells/well in 96-well plate, cultured for 24 h in an α-minimal essential medium (αMEM, Gibco, Grand Island, NY, USA) supplemented with 10% FBS, and then were treated with 100 ng/mL of recombinant murine soluble RANKL (Peprotech, Cranbury, NJ, USA) for 5 days to 7 days. Medium and drug were replaced every two days.

### 2.11. Tartrate-Resistant Acid Phosphate (TRAP) Assay

TRAP activity of differentiated osteoclast-like cells from RAW264.7 cells was determined using TRACP & ALP Assay Kit (Takara, Tokyo, Japan) and bicinchoninic acid (BCA) protein assay kits (Thermo Fisher Scientific, Waltham, MA, USA). After cells were differentiated, cells were washed once with physiological saline and then lysed by adding 60 μL of extraction solution. Cell lysates (50 μL) were incubated with equal volume of substrate solution, which contained 12.5 mM p-nitro-phenyl phosphate, 50 mM sodium tartrate and sodium acetate buffer (pH 5.2) for 1 h at 37 °C. The reaction was stopped by adding 50 μL of 0.5 N NaOH, and absorbance of the mixture was determined at 405 nm. Then TRAP specific activity (μU/μg) was calculated by normalization against protein concentration.

### 2.12. Protein Extraction and Nano-LC-ESI-MS/MS Analysis and Gene Ontology Analysis

Human aortic valve tissues were collected from three patients undergoing aortic valve replacement surgery at Asan Medical Center (Seoul, Republic of Korea). Calcified and adjacent non-calcified regions from each valve were separated and analyzed as paired samples without pooling for proteomic analysis. The study was approved by the Institutional Review Board of Asan Medical Center (IRB No. 2017-0556), and written informed consent was obtained from all patients.

Tissue samples were homogenized in 300 μL of 5% SDS in 50 mM NH_4_HCO_3_ using a bead beater (GeneResearch Biotechnology Co., Taiwan, China). Proteins were extracted from tissue lysates, reduced with 20 mM dithiothreitol in 50 mM NH_4_HCO_3_ for 10 min at 95 °C, and alkylated with 40 mM iodoacetamide in 50 mM NH_4_HCO_3_ for 30 min in the dark. S-TRAP™ (Protifi, New York, NY, USA) was used for the fast and reproducible preparation of samples, according to the manufacturer’s protocol and previous study [24]. Denatured proteins were bound to the S-TRAP™ and incubated overnight at 37 °C with 12.5 μg sequencing grade modified trypsin/LysC (Promega, Madison, WI, USA) in 50 mM NH_4_HCO_3_ buffer (pH 7.8). The eluted peptide samples were dried and quantified. The samples were resuspended in 0.1% formic acid and analyzed using an UltiMate 3000 RSLCnano system coupled to a Q Exactive™ Plus Hybrid Quadrupole-Orbitrap™ mass spectrometer with a Nano-ESI source (Thermo Fisher Scientific). Tryptic peptides from the bead column were reconstituted in 100 μL of 0.1% formic acid (FA) in water, and separated on an Acclaim™ Pepmap 100 C18 column (500 mm × 75 μm i.d., 3 μm, 100 Å) equipped with a C18 Pepmap trap column (20 mm × 100 μm i.d., 5 μm, 100 Å; Thermo Fisher Scientific) over 200 min (250 nL/min) using a 5–40% ACN gradient in 0.1% FA and 5% DMSO for 150 min (250 nL/min) at 50 °C. Mass spectra were acquired in data-dependent mode with an automatic switch between a full scan and the top 20 data-dependent MS/MS scans. The target value for the full-scan MS spectra, selected from 350 to 1800 *m*/*z*, was 3,000,000 AGS (Automatic gain control) targets, with a maximum injection time of 100 ms and a resolution of 70,000.

The obtained MS/MS spectra were assigned to proteins using the Sequest-HT on Proteome Discoverer (version 2.4, Thermo Fisher Scientific) and the UniProt human protein database (April 2024). Protein abundance data were imported into Perseus (version 2.0.7.0) for downstream statistical analyses. Missing values were imputed using the Replace missing values from normal distribution function with the following parameters: width = 0.3, down shift = 1.8, and mode = separately for each column. Principal component analysis (PCA), volcano plot analysis, and heatmap visualization were subsequently performed in Perseus. Differentially expressed proteins between paired normal and calcified valve tissues were identified using a two-sided paired Student’s *t*-test. Gene Ontology (GO) annotation and functional enrichment analysis were performed using ShinyGO version 0.77.

### 2.13. Statistical Analysis

All numerical values were expressed as mean ± standard deviation (SD). Multiple comparison tests for different dose groups were conducted. Variance homogeneity was examined using the Levene test [25]. If the Levene test indicated no significant deviation from variance homogeneity, the obtained data were analyzed by one-way ANOVA test followed by Tukey’s honest significant difference (THSD) test to determine which pairs of groups were significantly different. In case significant deviations from variance homogeneity were observed in the Levene test, Dunnett’s T3 (DT3) test was executed to determine which pairs of groups comparison were significantly different [26,27]. Differences were considered significant at *p* < 0.05. Statistical analyses were conducted using SPSS for Windows (Release 18.0, SPSS Inc., Chicago, IL, USA). In addition, the percentage changes were calculated to monitor the severities of CAVD and ectopic calcification with related organ damage and to help the understanding of efficacy, working in this model, as follows in Equations (1) and (2), according to previously established method [28,29].

Percentage changes as compared with normal vehicle control (%) =[((Data of vitamin D3 vehicle control − Data of normal vehicle control mice)/Data of normal vehicle control mice) × 100](1)

Percentage changes as compared with vitamin D3 vehicle control (%) =[((Data of test article treated mice − Data of Vitamin D3 vehicle control mice)/Data of Vitamin D3 vehicle mice) × 100](2)

## 3. Results

### 3.1. TNAP Is Overexpressed in CAVD

**Figure 1:**
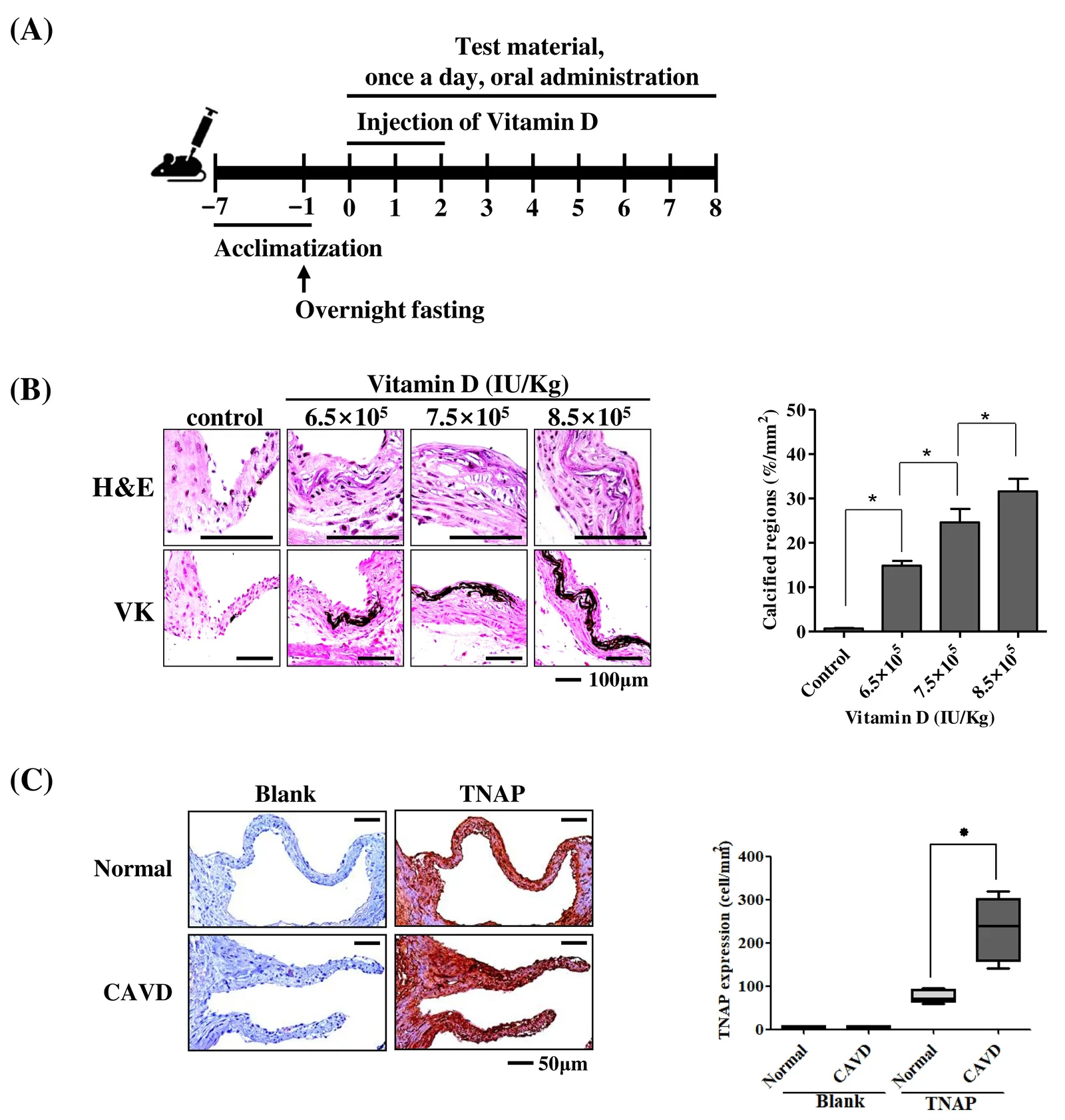
TNAP is overexpressed in CAVD-induced mouse model. (**A**) Schematic representation of vitamin D3-induced CAVD mouse model. (**B**) H&E and von Kossa (VK) staining of aortic valve tissue sections from normal and vitamin D_3_-induced CAVD mice at the indicated doses (6.5 × 10^5^, 7.5 × 10^5^, 8.5 × 10^5^ IU/kg) (scale bar = 100 μm). (Calcified areas are stained in black) (**C**) TNAP expression in normal and CAVD mouse valve tissues, assessed by immunohistochemical staining and quantitative analysis (scale bar = 50 μm). (Brown indicates positive staining; blue indicates cell nuclei counterstained with hematoxylin). Data are shown as mean ± SD. * *p*-value < 0.05.

CAVD mouse model was established by administering vitamin D_3_ via subcutaneous injection once daily for 3 days, followed by an additional 5-day induction period (Figure 1A). For observing the CAVD induction, mice hearts were isolated, and the aortic valve region was sectioned for H&E and von Kossa staining. Given the pathological characteristics of CAVD, histological and molecular analyses were systematically evaluated across the entire aortic valve complex, encompassing both the valve leaflets and the surrounding basal microenvironment. The thickness of aortic valves was increased, and calcified regions were significantly elevated in dose-dependent manner following vitamin D treatment (*p* < 0.01, *p* < 0.0001, and *p* < 0.0001, respectively) (Figure 1B). We hypothesized that TNAP represents a potential therapeutic target in CAVD. Consistent with its physiological function, TNAP was detectable in the normal aortic valve. However, upon CAVD induction by vitamin D, TNAP expression was markedly upregulated in the aortic valve tissue indicating its pathological involvement in disease progression (*p* < 0.05, *N* = 5) (Figure 1C). Collectively, these data confirm successful induction of CAVD by excessive vitamin D administration and demonstrate that TNAP expression is markedly increased in calcified aortic valves.

### 3.2. Proteomic Profiling

**Figure 2:**
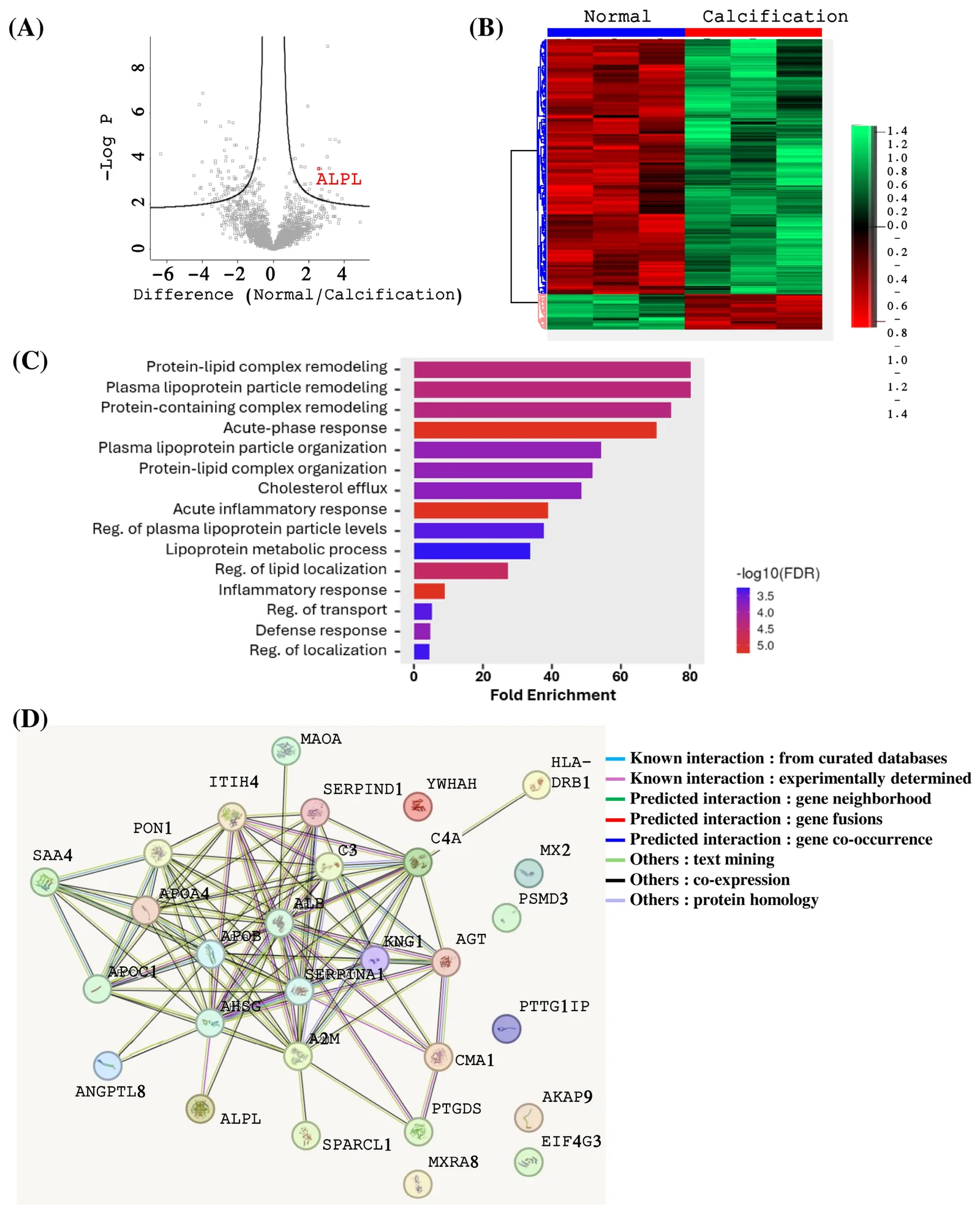
Proteomics analysis of calcified human aortic valve tissues. (**A**) Volcano plot between normal and calcified aortic valve tissues. (**B**) Heatmap of 277 proteins. (**C**) Gene Ontology enrichment analysis of the 34 up-regulated proteins. (**D**) Protein–protein interaction network of the 34 up-regulated proteins generated using the STRING database.

To comprehensively understand the molecular mechanism of calcification, we conducted proteomic analysis using the aortic valve tissues of CAVD patients. Volcano plot demonstrates an overview of the differential expression of the proteins between normal and calcified tissues (Figure 2A) and a separation between the two groups was revealed by principal component analysis (PCA). A total of 277 proteins, which had Student’s *t*-test with a Permutation-based FDR of <0.05, were selected and z-score applied to spectral counts was used to perform heatmap (Figure 2B). In calcified regions, 34 proteins were up-regulated and 243 proteins were down-regulated; notably, TNAP (encoded by ALPL) was included among the up-regulated proteins (Supplementary Table S2). Consistently, ALPL expression was also increased in osteogenic VICs based on NGS data analysis (GSE252266) (Supplementary Figure S3A).

Gene Ontology (GO) annotation and functional enrichment analysis were performed using ShinyGO 0.85.1 (http://bioinformatics.sdstate.edu/go/) (accessed on 26 November 2025). The 277 differentially expressed proteins were mainly associated with metabolic processes (Supplementary Figure S3B), and the 34 up-regulated proteins were enriched in lipid metabolism-related terms and inflammatory response-related categories (Figure 2C). Protein–protein interaction (PPI) analysis of the 34 up-regulated proteins was performed using the STRING database to assess known and predicted interactions (Figure 2D). Several proteins formed interconnected clusters within the network, while other proteins appeared as isolated or weakly connected nodes. ALPL was included among the mapped proteins in the STRING network.

### 3.3. Inhibition of TNAP Reduces Calcification

**Figure 3:**
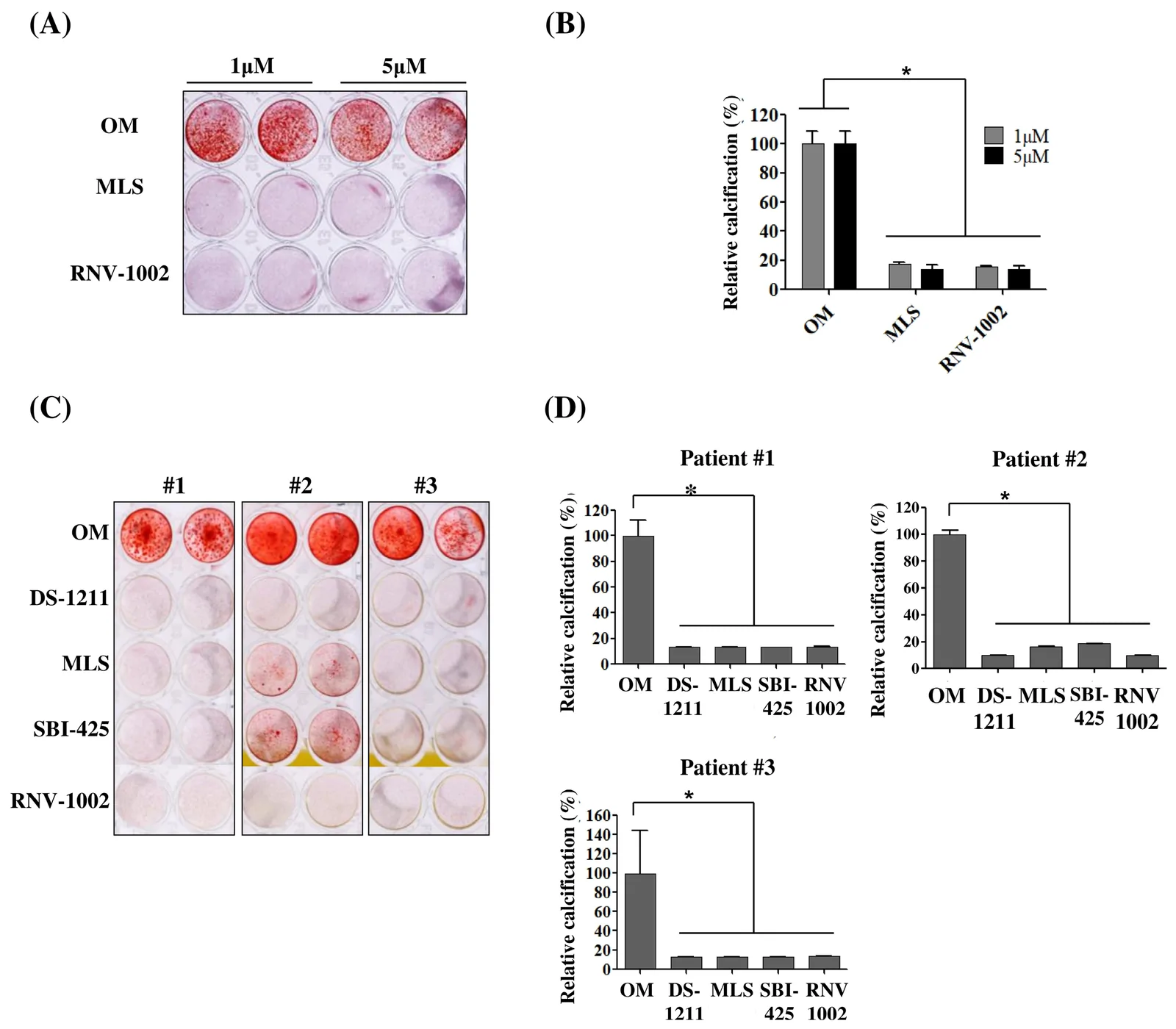
Effects of TNAP inhibitors in patients-derived VICs. (**A**) Alizarin Red-S staining in SaOS-2 cells. (**B**) Quantitative analysis of calcification in SaOS-2. (**C**) Alizarin Red-S staining in VICs from 3 individual patients. (**D**) Quantitative analysis of calcification in VICs. (OM, osteogenic medium). * *p*-value < 0.05.

We examined the effect of TNAP inhibition on calcification using SaOS-2 cells and patient-derived VICs. VICs were isolated from human aortic valves obtained during surgical replacement for CAVD (Supplementary Figure S1A). To date, only three TNAP inhibitors have been reported: MLS0038949, SBI-425, and DS-1211. To expand this limited repertoire, we developed a novel series of TNAP inhibitors and identified compound #178 as the most potent and effective among them, subsequently naming it RNV-1002. RNV-1002 demonstrated the greatest efficacy in both SaOS-2 cells and patient-derived VICs (Supplementary Figure S1B–E), and exhibited the most potent inhibitory activity, with an IC_50_ of 6.5 nM (Supplementary Table S1). TNAP inhibitors, including MLS0038949 and RNV-1002, inhibit the calcification in SaOS-2 cells with 1 μM and 5 μM treatment (Figure 3A,B). To confirm the efficacy, we used three different patient-derived VICs and TNAP inhibition showed about 90% inhibition of calcification by 1 μM treatment (Figure 3C,D).

Furthermore, to address whether TNAP inhibition can attenuate ongoing calcification rather than merely preventing its onset, we performed delayed-treatment experiments in human VICs undergoing a 28-day osteogenic induction protocol. TNAP inhibitors (MLS-0038949 and SBI-425) were introduced at delayed time points after induction (Days 0, 7, 10, 14, and 21). Delayed administration of these inhibitors significantly suppressed calcium deposition in a time-dependent manner, even when introduced mid-course during active osteogenesis (Supplementary Figure S4). These findings suggest that targeting TNAP can effectively slow down and attenuate ongoing calcification cascades, demonstrating both preventive and therapeutic potential beyond initial disease prevention.

### 3.4. Effects of TNAP Inhibitors on Calcification in a CAVD Mouse Model

**Figure 4:**
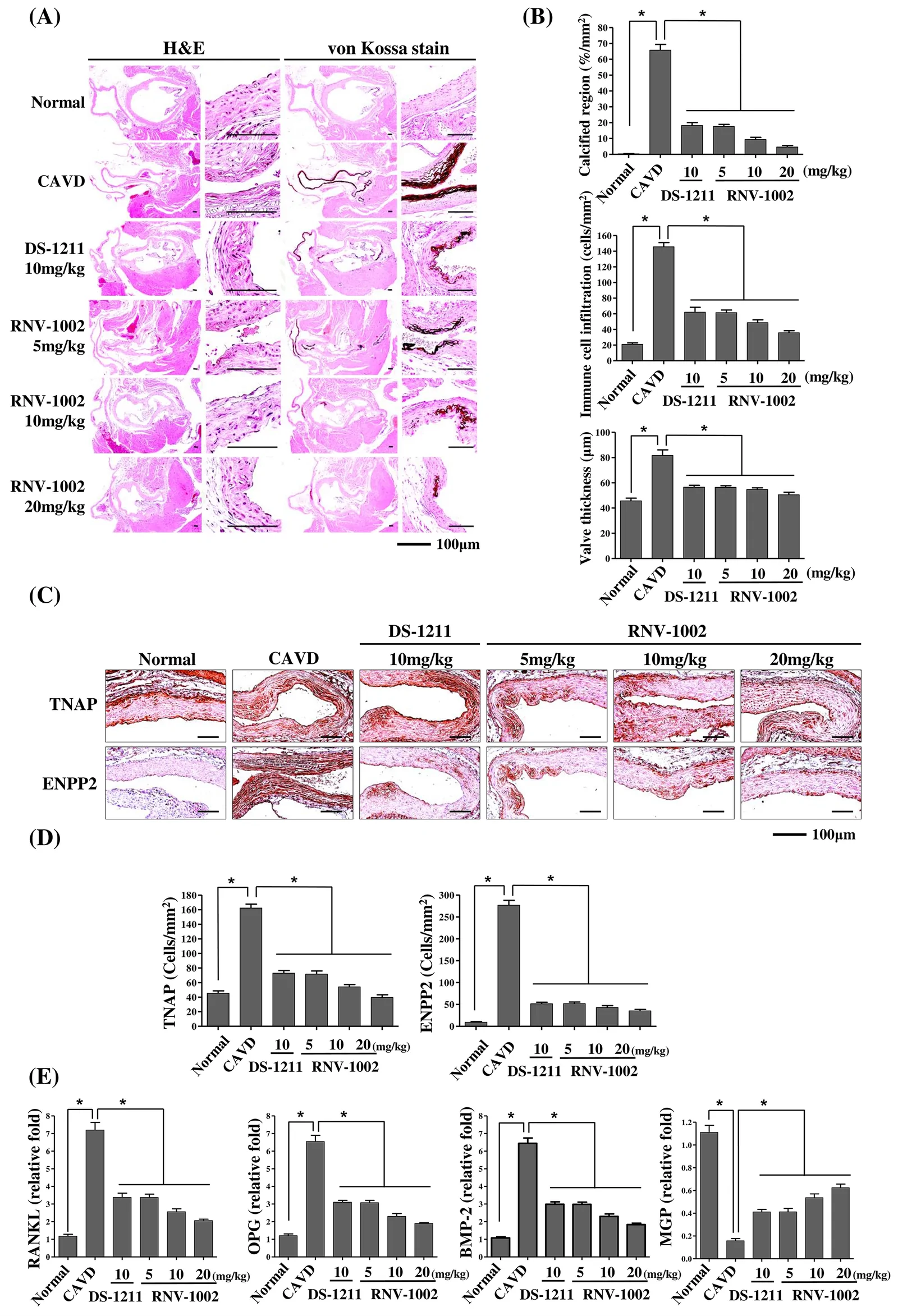
Effects of TNAP inhibitors on calcification in a CAVD mouse model. (**A**) H&E and von Kossa staining of aortic valve tissue from normal, CAVD, DS-1211 treated, and RNV-1002 treated mice (DS-1211 at 10 mg/kg; RNV-1002 at 5, 10, and 20 mg/kg) (Scale bar = 100 μm). (Calcified areas are stained in black) (**B**) Quantitative analysis of annulus thickness, immune cell infiltration, and calcified regions, respectively. (**C**) Immunohistochemical staining of aortic valves for TNAP and ENPP2, respectively (Scale bar = 100 μm). (Brown indicates positive staining; blue indicates cell nuclei counterstained with hematoxylin) (**D**) Quantification of TNAP and ENPP2-positive cells per mm^2^ of the aortic valve region across the indicated groups. (**E**) Relative mRNA expression levels of pro-osteogenic markers (*RANKL*, *OPG*, and *BMP-2*) and an anti-calcific marker (*MGP*) in heart tissue. Data are shown as mean ± SD. * *p*-value < 0.05.

To evaluate the validity of TNAP as a therapeutic target, vitamin D-induced CAVD mice were orally administered RNV-1002 (5, 10, or 20 mg/kg) or the reference TNAP inhibitor DS-1211 (10 mg/kg) once daily for 8 days (*N* = 10 in each group). Histological analysis using hematoxylin and eosin (H&E) and von Kossa staining revealed severe leaflet thickening and extensive calcium deposition in the aortic valves of CAVD mice compared with normal controls (Figure 4A). Treatment with either RNV-1002 or DS-1211 significantly reduced valve thickness, inflammation, and calcification. Valve thickness was reduced to levels similar to normal and inflammation and calcification reduced dose-dependent manner (Figure 4A,B). Treatment with TNAP inhibitors markedly suppressed the expression of both TNAP and ENPP2 in a dose-dependent manner (Figure 4C). Quantitative analysis confirmed that the numbers of TNAP- and ENPP2-positive cells were significantly reduced (Figure 4D, *p* < 0.0001). mRNA expression of pro-osteogenic factors, including receptor activator of nuclear factor-κB ligand (*RANKL*), bone morphogenetic protein 2 (*BMP2*), and osteoprotegerin (*OPG*) was increased in CAVD mouse hearts and decreased following TNAP inhibitor treatment. In contrast, expression of matrix Gla protein (*MGP*), a known inhibitor of calcification, was downregulated in CAVD mice but restored by TNAP inhibitors. All responses to RNV-1002 occurred in a dose-dependent pattern (Figure 4E). Taken together, these findings implicate TNAP as a critical molecular target in CAVD, as blocking its activity simultaneously downregulates key pro-osteogenic signaling pathways and rescues protective matrix-stabilizing factors.

### 3.5. Regulation of TNAP Affects Oxidative Stress

Various pathological and signaling pathways have been implicated in CAVD progression, although the underlying mechanisms remain incompletely understood. Oxidative stress, lipid peroxidation, inflammation, apoptosis, and fibrosis are well recognized molecular features associated with valvular calcification. To experimentally verify these pathological changes, we examined these processes in vitamin D-induced CAVD mouse model.

**Figure 5:**
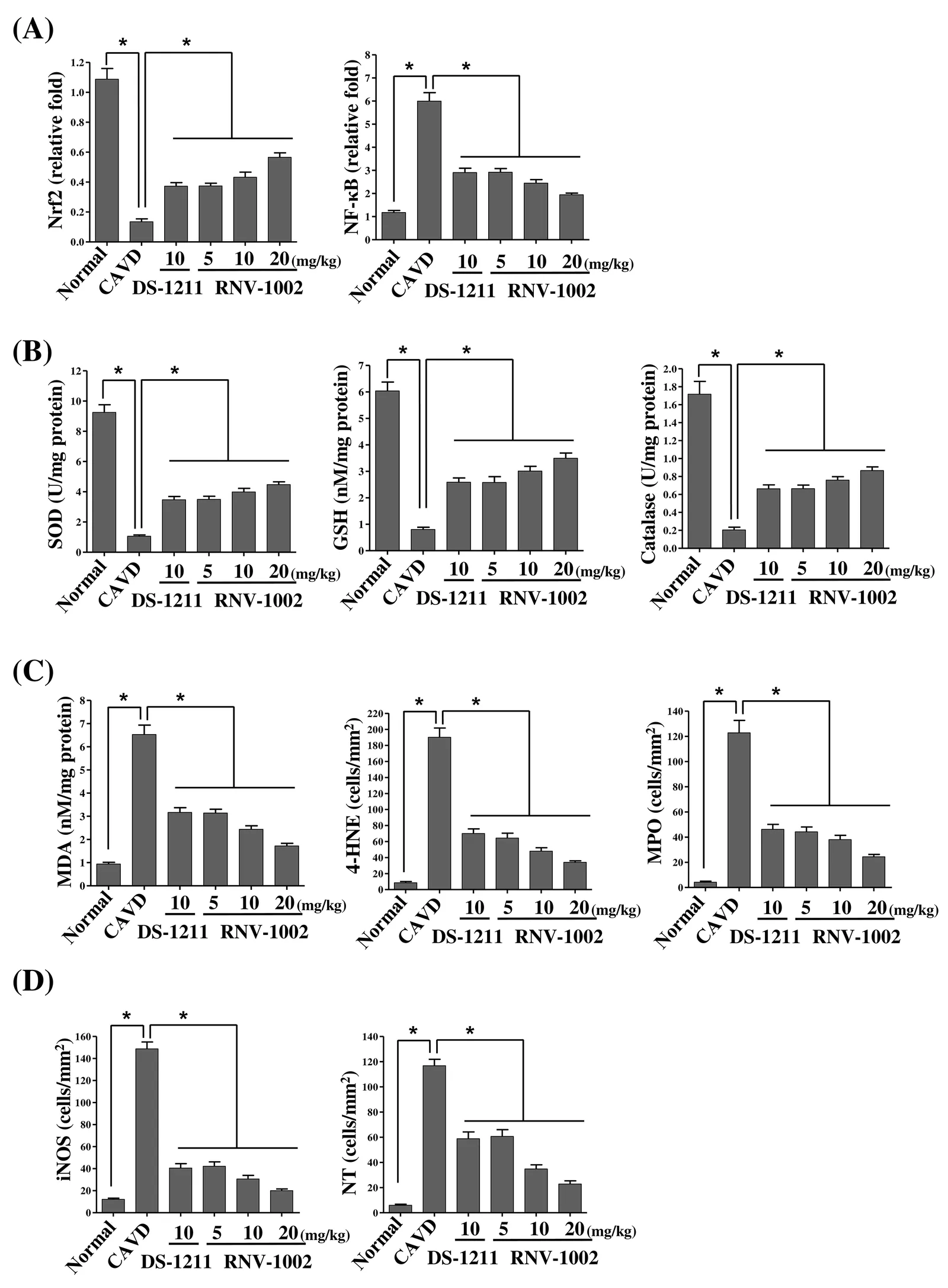
Effects of TNAP inhibitors on oxidative stress, lipid peroxidation, and RNS signaling. (**A**) mRNA level of ROS response markers (*Nrf2* and *NF-κB*). (**B**) Activity levels of antioxidant markers (SOD, GSH and CAT) in heart tissue. (**C**) Levels of lipid peroxidation markers (MDA, 4-HNE, and MPO) in heart tissue. (**D**) RNS signaling markers (iNOS and NT) in heart tissue. * *p*-value < 0.05.

Nuclear factor erythroid2-related factor 2 (*Nrf2*), a transcription factor associated with antioxidant response, was decreased in CAVD model, whereas nuclear factor kappa-light-chain-enhancer of activated B cell (*NF-κB*), a key regulator of inflammation, was increased. These alterations were reversed by TNAP inhibitor treatment (Figure 5A). Levels of oxidative stress-related antioxidants, such as glutathione (GSH), superoxide dismutase (SOD), and catalase (CAT), were markedly reduced in CAVD model, whereas treatment with TNAP inhibitors restored these antioxidant levels in a dose-dependent manner (Figure 5B). Lipid peroxidation markers, malondialdehyde (MDA), 4-hydroxy-2-nonenal (4-HNE), myeloperoxidase (MPO), were elevated in hearts of CAVD mice, and these increases were attenuated following TNAP inhibitor treatment (Figure 5C). RNS signaling markers, inducible nitric oxide synthase (iNOS) and 3-nitrotyrosine (3-NT), were all significantly upregulated in CAVD-induced mice and were reduced by administration of TNAP inhibitors (Figure 5D).

### 3.6. Inhibition of TNAP Attenuates Inflammation, Apoptosis and Fibrosis

**Figure 6:**
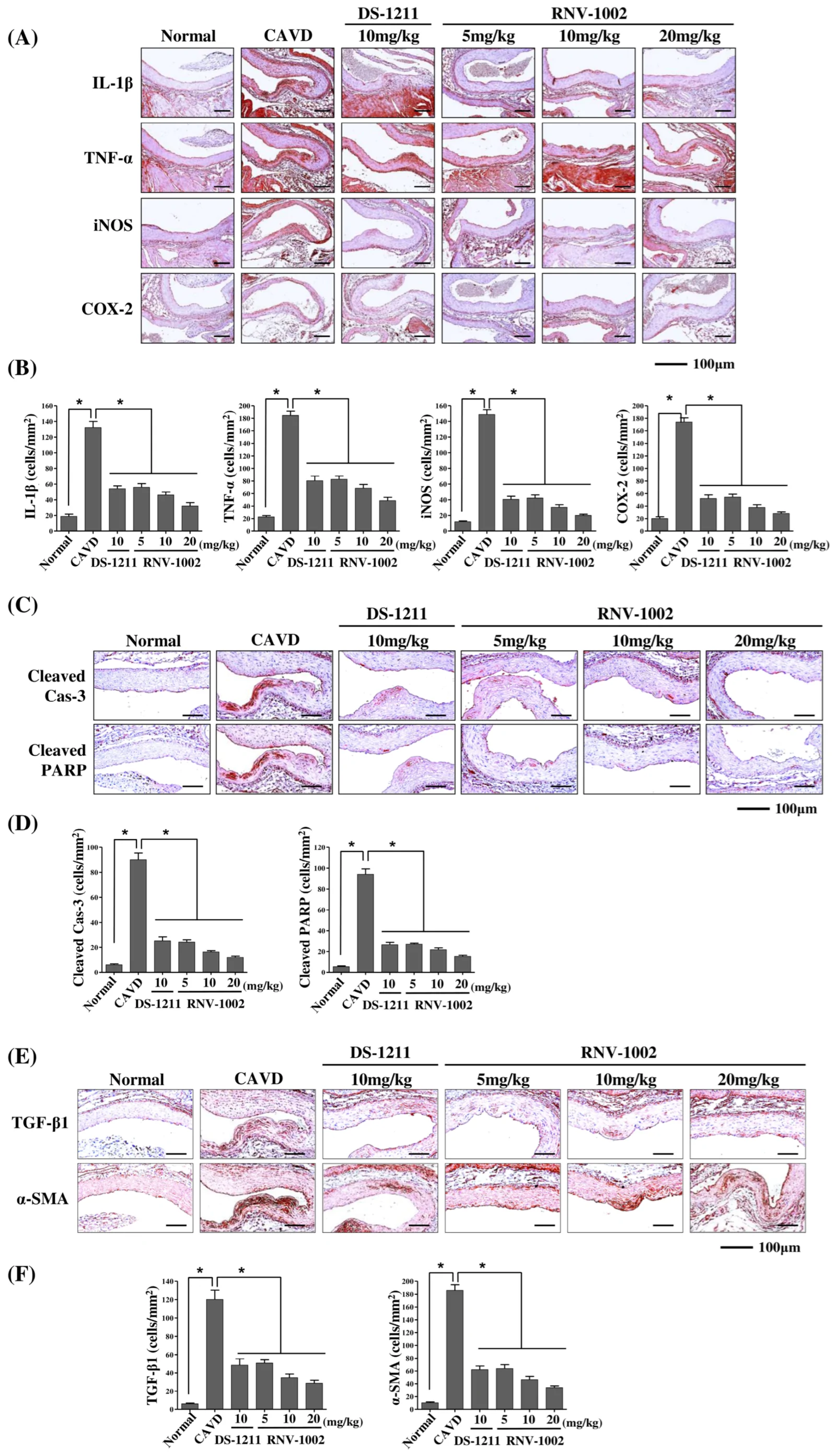
Effects of TNAP inhibitors on inflammation, apoptosis and fibrosis in a CAVD mouse model. (**A**,**B**) Immunohistochemical staining and quantitative analysis of IL-1β, TNF-α, iNOS, and COX-2 in aortic valve tissues (Scale bar = 100 μm). (**C**,**D**) Immunohistochemical staining and quantitative analysis of Cleaved Cas-3 and Cleaved PARP in aortic valve tissues (Scale bar = 100 μm). (**E**,**F**) Immunohistochemical staining and quantitative analysis of fibrosis markers TGF-β1 and α-SMA in aortic valve tissues. Data are shown as mean ± SD. * *p*-value < 0.05.

To assess inflammation response, we measured representative pro-inflammatory mediators. Interleukine-1beta (IL-1β), tumor necrosis factor-alpha (TNF-α), inducible nitric oxide synthase (iNOS), and cyclooxygenase-2 (COX-2) were all significantly upregulated in CAVD-induced mice and were reduced by TNAP inhibitor treatment (Figure 6A,B). In the middle and late stages of CAVD, the aortic valve exhibited clear signs of apoptosis and fibrotic remodeling. Cleaved caspase-3 (Cas-3) and cleaved PARP, two key markers of apoptotic activation, were substantially elevated in CAVD mice, indicating increased cell death within the valve tissue. These changes were notably reduced following treatment with TNAP inhibitors (Figure 6C,D). Because apoptotic signaling often triggers extracellular matrix remodeling [30,31], we next assessed fibrosis-related markers. Expression of TGF-β1 and α-SMA, both involved in myofibroblast activation and fibrotic progression, was increased in the hearts of CAVD mice. Administration of TNAP inhibitors attenuated these elevations, suggesting that suppression of TNAP activity helps limit fibrotic remodeling of the valve (Figure 6E,F). Together, these findings indicate that TNAP inhibition not only reduces apoptosis but also suppresses downstream fibrotic changes that contribute to valvular degeneration during CAVD progression.

### 3.7. Effects of RNV-1002 on Osteoclastogenesis and Cytotoxicity

Inhibition of TNAP has been associated with reduced ectopic calcification, raising concerns about potential effects on physiological bone formation, including the risk of osteoporosis. Because osteoclastogenesis and osteoblastogenesis function in a coordinated manner during bone remodeling, we examined whether TNAP inhibition influences osteoclast differentiation.

**Figure 7:**
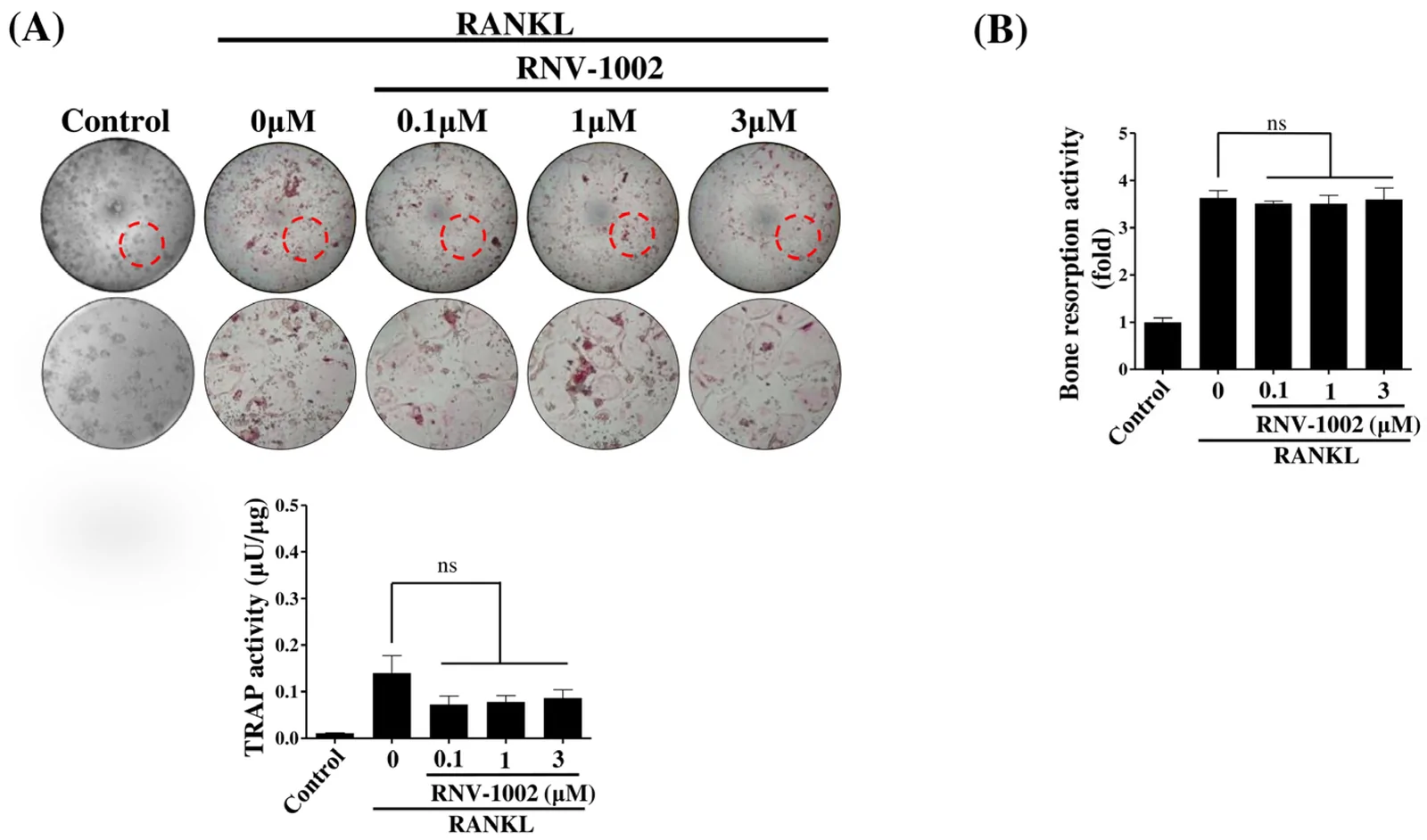

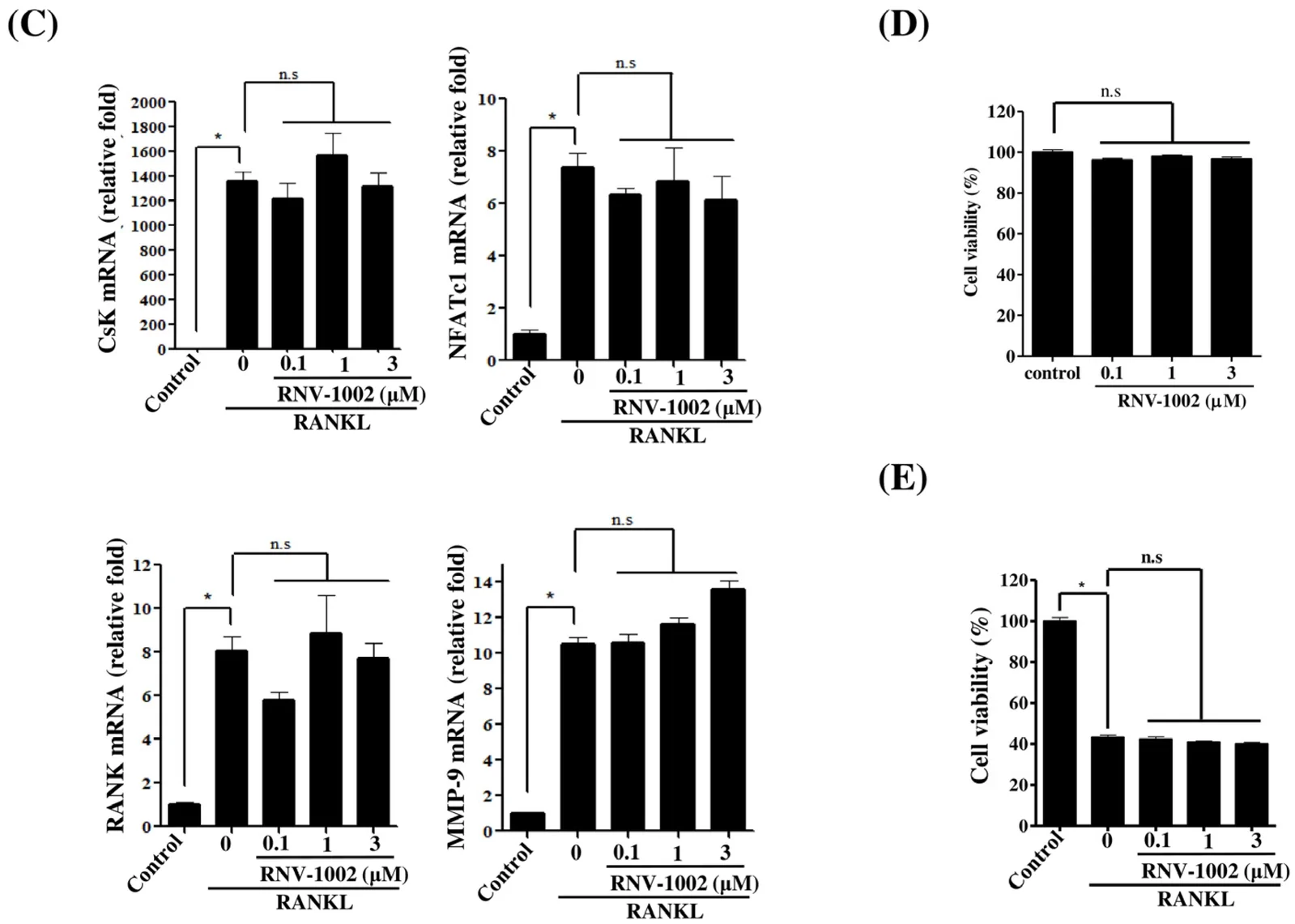
Effects of RNV-1002 on in vitro osteoclastogenesis. (**A**) TRAP staining and quantitative analysis of osteoclast-like cell differentiation in RANKL-induced osteoclasts treated with RNV-1002. The lower row of images represents magnified views of the areas highlighted by the red circles in the upper row. (**B**) Relative fluorescence intensity of RANKL-induced osteoclasts treated with RNV-1002. (**C**) Relative mRNA expression levels of osteoclast differentiation markers (*CsK*, *NFATc1*, *RANK* and *MMP-9*) in RANKL-induced osteoclasts treated with RNV-1002. (**D**) Cell viability of RAW264.7 cells treated with RNV-1002. (**E**) Cell viability of RANKL-induced osteoclasts treated with RNV-1002. Data are shown as mean ± SD. * *p*-value < 0.05, ns: not significant.

RAW264.7 cells were induced to differentiate into osteoclast-like cells by RANKL treatment for five days. During this process, RANKL-stimulated cells were co-treated with RNV-1002 (0.1, 1, and 3 μM). TRAP activity was comparable between RANKL-induced cells with or without RNV-1002 treatment, indicating that TNAP inhibition did not affect osteoclast differentiation or activation (Figure 7A). To further assess the functional capacity of osteoclasts, bone resorption activity was measured using a fluoresceinamine-labeled chondroitin sulfate and calcium phosphate-coated substrate. RNV-1002 treatment did not alter the fluorescence intensity of conditioned medium relative to the control group (Figure 7B). We next evaluated genes associated with osteoclast differentiation, including nuclear factor of activated T cells cytoplasmic 1 (*NFATc1*), c-src tyrosine kinase (*CsK*), matrix metalloproteinase-9 (*MMP-9*), and *RANK*. However, cotreatment with RNV-1002 (0.1–3 μM) did not modify RANKL-induced expression of any of these genes (Figure 7C). Cell viability assays further confirmed that RNV-1002 had no cytotoxic effects on either Raw264.7 cells (Figure 7D) or RANKL-induced osteoclast-like cells (Figure 7E). Thus, the absence of changes in osteoclast differentiation markers or resorptive activity indicates that TNAP inhibition by RNV-1002 does not engage osteoclast-related pathways. To evaluate the potential effects of RNV-1002 on skeletal homeostasis, whole-body dual-energy X-ray absorptiometry (DEXA) and histological analyses were conducted in normal mice treated with 100, 200, or 400 mg/kg RNV-1002 for 28 days. DEXA analysis showed no significant differences in bone mineral density (BMD) of the total body, left femur, or calvaria among RNV-1002–treated groups compared with the control (Supplementary Figure S2).

## 4. Discussion

CAVD remains a major unmet clinical challenge, because no pharmacological therapy has yet been shown to effectively delay or reverse valvular calcification. CAVD that is regulated by spatially and temporal biological networks is a complex disease, and its pathogenesis is still unclear [32]. In this study, we demonstrate that TNAP is markedly upregulated in calcified aortic valves and that pharmacological inhibition of TNAP using a newly developed small-molecule inhibitor, RNV-1002, significantly attenuates calcification in both human VICs and a mouse model of CAVD.

Human VICs play an important role in the valve calcification process and serve as a useful in vitro model for studies of molecular mechanisms and drug efficacy [33]. To assess donor-to-donor variability, TNAP inhibitors were evaluated using VICs derived from three different individuals. TNAP inhibitors consistently reduced calcification across all three VIC lines with comparable efficacy. These findings suggest that TNAP functions as a primary driver of calcification across primary cells, which aligns with our in vivo observations showing reproducible anti-calcific effects with minimal inter-individual variability (low SD values) in animal models. This consistency supports the rationale for targeting TNAP as a therapeutic strategy in CAVD, although expanding human donor cohorts will further reinforce target engagement across broader clinical backgrounds.

As standard mouse models for calcific aortic valve disease (CAVD) have not been fully established, we employed a vitamin D-induced CAVD mouse model that has been used in previous studies of CAVD pathophysiology. Although genetic mutant models combined with high-fat diets can induce aortic valve calcification, such approaches may introduce confounding effects related to genetic background, complicating interpretation of therapeutic efficacy in the context of heterogeneous disease progression. Similarly, high-cholesterol diet models are based on the assumption that hyperlipidemia and atherosclerosis are primary drivers of CAVD [34], which does not fully reflect the multifactorial condition of valvular calcification. In contrast, vitamin D-induced CAVD models have been shown to reproduce key pathological features, including aortic stenosis with increased transvalvular peak jet velocity and pressure gradients, as well as fibrosis, marked valve calcification, and elevated osteogenic markers [35,36]. Moreover, vitamin D–induced CAVD has also been described in rabbit models, which exhibit aortic valve calcification and sclerosis resembling key aspects of human CAVD pathology [37]. In vivo, oral administration of RNV-1002 significantly attenuated valvular thickening, inflammation, and calcium deposition in vitamin D-induced CAVD mice in a dose-dependent manner. Notably, TNAP inhibition was accompanied by reduced expression of ENPP2 (autotaxin), a molecule previously implicated in valvular inflammation and fibrosis. This dual suppression suggests that TNAP inhibition may exert broader regulatory effects beyond mineral metabolism, potentially modulating inflammatory and fibrotic signaling pathways that contribute to disease amplification during CAVD progression. Consistent with this notion, inhibition of TNAP restored antioxidant defenses and reduced oxidative stress markers in CAVD mice. Oxidative stress and lipid peroxidation are recognized contributors to VIC activation and osteogenic reprogramming, and their attenuation likely contributes to the observed anti-calcific effects. Furthermore, TNAP inhibition suppressed pro-inflammatory mediators, including IL-1β, TNF-α, iNOS, and COX-2, while upregulating Nrf2 signaling and suppressing NF-κB activation. These findings suggest that TNAP inhibition indirectly mitigates inflammatory and oxidative cascades that reinforce valvular degeneration. Nevertheless, although the vitamin D-induced model is known to capture functional hemodynamic changes, a limitation of the present study is that direct in vivo functional evaluations—such as echocardiographic measurements of transvalvular peak velocity and pressure gradients—were not performed. Further translational studies incorporating echocardiography are warranted to confirm whether the observed histological and molecular improvements directly translate into hemodynamically functional recovery.

Advanced CAVD is characterized by extensive hydroxyapatite deposition and osteogenic signaling [38,39,40]. In line with this, pro-osteogenic markers such as RANKL and BMP2 were upregulated in CAVD hearts and markedly reduced by RNV-1002 treatment, while the calcification inhibitor MGP was restored. Together, these results indicate that TNAP inhibition shifts the valvular microenvironment away from a pro-mineralization state toward one that favors calcification suppression.

Beyond calcification, CAVD progression involves apoptosis and fibrotic remodeling, which contribute to irreversible structural decay of the valve [41,42,43]. TNAP inhibitors significantly reduced cleaved caspase-3 and PARP levels, indicating attenuation of apoptosis within valve tissue. In parallel, reduced expression of TGF-β1 and α-SMA suggests suppression of myofibroblast activation and fibrotic remodeling. These effects likely reflect secondary benefits of TNAP inhibition arising from reduced calcific stress and inflammatory signaling within the valve. Given the essential role of TNAP in physiological bone mineralization, safety concerns regarding skeletal homeostasis are of particular importance [16]. Our data showed that RNV-1002 does not affect osteoclast-like cell differentiation, resorptive activity, or viability in vitro. Moreover, long-term high-dose administration of RNV-1002 in normal mice did not alter bone mineral density, trabecular or cortical bone architecture, or osteoclast parameters in either long bones or flat bones. These findings suggest that selective TNAP inhibition can effectively target pathological calcification without disrupting normal bone remodeling under the tested conditions.

Several limitations should be acknowledged. The vitamin D-induced CAVD model recapitulates key features of valvular calcification but may not fully reflect the chronic and multifactorial nature of human disease. In addition, longer treatment durations and studies in large-animal models will be necessary to further establish the translational potential of TNAP inhibition. Nevertheless, the consistent anti-calcific, anti-inflammatory, and anti-fibrotic effects observed across in vitro and in vivo systems strongly support TNAP as a viable therapeutic target.

In conclusion, this study demonstrates that pharmacological inhibition of TNAP using the novel inhibitor RNV-1002 effectively attenuates valvular calcification and associated pathological remodeling without compromising bone homeostasis. These findings provide compelling preclinical evidence supporting TNAP inhibition as a promising therapeutic strategy for the treatment of CAVD.

## Figures and Tables

**Table 1:** Primer sequences.

Gene	Forward Primer	Reverse Primer
***RANKL***	5′-GTGAAGACACACTACCTGACTCC-3′	5′-GCCACATCCAACCATGAGCCTT-3′
***OPG***	5′-CGGAAACAGAGAAGCCACGCAA	5′-CTGTCCACCAAAACACTCAGCC-3′
***BMP2***	5′-AACACCGTGCGCAGCTTCCATC-3′	5′-CGGAAGATCTGGAGTTCTGCAG -3′
***MGP***	5′-AGGAACGCAACAAGCCTGCCTA-3′	5′-CTGCCTGAAGTAGCGGTTGTAG-3′
***Nrf2***	5′-CAGCATAGAGCAGGACATGGAG-3′	5′-GAACAGCGGTAGTATCAGCCAG-3′
***NF-κB***	5′-GCTGCCAAAGAAGGACACGACA-3′	5′-GGCAGGCTATTGCTCATCACAG-3′
***β-actin***	5′-GTGAAGACACACTACCTGACTCC-3′	5′-TGCTGGAAGGTGGACAGTGAGG-3′

**Table 2:** Primary antibody and detection kits used in immunochemistry.

Antibodies and Detection Kits	Source	Dilution
**Primary antibody**		
Anti-ALPL antibody	ABclonal, Woburn, MA, USA	1:100
Anti-ENPP2 polyclonal antibody	Bioss, Woburn, MA, USA	1:200
Anti-cleaved caspase-3 antibody	Cell Signaling Technology Inc., Danvers, MA, USA	1:200
Anti-cleaved PARP(h215) antibody	Santa Cruz Biotechnology Inc., Dallas, TX, USA	1:200
Anti-MPO antibody	ABclonal, Woburn, MA, USA	1:100
Anti-NOS2(N-20) antibody	Santa Cruz Biotechnology, Dallas, TX, USA	1:100
Anti-COX-2 antibody	Cayman Chemical, Ann Arbor, MI, USA	1:200
Anti-TNF-α(4E1) antibody	Santa Cruz Biotechnology Inc., Dallas, TX, USA	1:100
Anti-IL-1β(H153) antibody	Santa Cruz Biotechnology Inc., Dallas, TX, USA	1:100
Anti-NT polyclonal antibody	Millipore, St. Louis, MO, USA	1:200
Abti-4-HNE polyclonal antibody	Abcam, Cambridge, UK	1:100
Anti-TGF-β1(3C11) antibody	Santa Cruz Biotechnology, Dallas, TX, USA	1:100
Anti-α-SMA(ACTA2) antibody	ABclonal, Woburn, MA, USA	1:100
**Detection kits**		
Vectastain Elite ABC kit	Vector Lab., Newark, CA, USA	1:50
Peroxidase substrate kit	Vector Lab., Newark, CA, USA	1:100

All antibodies were diluted using 0.01 M phosphate buffered saline (pH 7.2).

## Data Availability

The original contributions presented in this study are included in the article/Supplementary Material. Further inquiries can be directed to the corresponding author.

## Supplementary Materials

The following supporting information can be downloaded at: https://www.mdpi.com/article/10.3390/cells15171543/s1, Figure S1: Efficacy of novel TNAP inhibitors in VICs and SaOS-2 cells. (A) Gross morphology of aortic valve tissues obtained from CAVD patients. (B) Alizarin Red S staining of candidate TNAP inhibitors in patient-derived VICs. (C) Quantitative analysis of relative calcification levels in patient-derived VICs. (D) Alizarin Red S staining of candidate TNAP inhibitors in SaOS-2 cells. (E) Quantitative measurement of relative calcification levels in SaOS-2 cells; Figure S2: Effects of RNV-1002 on bone. (A) DEXA images of whole-body scans from control, RNV-1002–treated mouse (100, 200, and 400 mg/kg). (B) Quantitative analysis of BMD in total body, left femur, and calvaria from control, RNV-1002–treated mouse. (C) H&E staining in femur from control, RNV-1002–treated mouse (Scale bar = 80 μm). (D) Quantitative analysis of bone parameters, including TBV, Cbt, Ocn, OC/BS from control, RNV-1002–treated mouse. Data are shown as mean ± SD. * *p*-value < 0.05; Figure S3: NGS analysis of VICs with/without CAVD. (A) Heatmap of ALPL expression in osteogenic valvular interstitial cells (VICs) analyzed using NGS data (GSE252266). (B) Gene Ontology annotation of 277 genes identified from gene expression analysis; Figure S4: Delayed TNAP inhibition attenuates ongoing calcification in primary human valve interstitial cells (hVICs). Human VICs were cultured in osteogenic medium (OM) for 28 days to induce calcification. TNAP inhibitors were added at indicated post-induction time points (Day 0, 7, 10, 14, and 21) to evaluate the therapeutic potential on ongoing osteogenesis. (A) Representative Alizarin Red S (ARS) staining images showing the effect of delayed treatment with MLS-0038949 (10 μM). (B) Spectrophotometric quantification of ARS staining shown in (A). (C) Representative ARS staining images showing the effect of delayed treatment with SBI-425 (10 μM). (D) Spectrophotometric quantification of ARS staining shown in (C). Data are presented as mean ± SD (n = 8 biological replicates); Table S1: IC_50_ of TNAP inhibitors; Table S2: The protein expression list by proteomic analysis. Reference [44] is cited in Supplementary Materials.

## Abbreviations

CAVD	Calcific aortic valve disease
VICs	Valvular interstitial cells
TNAP	Tissue non-specific alkaline phosphatase
H&E	Hematoxylin and Eosin staining
VK	von Kossa staining
TRAP	Tartrate-resistant acid phosphatase
NFATc1	Nuclear factor of activated T cells, cytoplasmic 1
RANKL	Receptor activator of NF-κB ligand
CsK	c-src tyrosine kinase
MMP-9	Matrix metalloproteinase-9
ENPP2	Ectonucleotide pyrophosphatase/phosphodiesterase 2
GSH	Glutathione
SOD	Superoxide dismutase
CAT	Catalase
IL-1β	interleukin 1-beta
TNF-α	Tumor necrosis factor-alpha
iNOS	inducible nitric oxide synthase
COX-2	Cyclooxygenase
MDA	Malondialdehyde
4-HNE	4-Hyfroxynonenal
MPO	Myeloperoxidase
Cas-3	Caspase 3
PARP	poly(ADP-ribose) polymerase
TGF-β1	Tumor growth factor-beta 1
α-SMA	alpha-Smooth muscle actin
OPG	Osteoprotegerin
BMP-2	Bone morphogenetic protein 2
MGP	Matrix Gla protein
Nrf2	Nuclear factor erythroid 2-related factor 2
NF-κB	Nuclear factor kappa-light-chain-enhancer of activated B cell
DEXA	Dual-energy x-ray absorptiometry
Cb	Cortical bone
Tb	Trabecular bone
Bm	Bone marrow
Gp	Growth plate
BV	Bone volume
TBV	Trabecular bone volume
Cbt	Thickness of the femur cortical bone
Pbt	Thickness of the calvaria plate
Ocn	Osteoclast cell number
OC/BS	Osteoclast call ratios on the bone surface
