# Supplementary material for: Inhibiting TNAP Attenuates the Aortic Valve Calcification and Reduces Oxidative Stress
Source: Cells. 2026 Aug 26;15(17):1543. doi: 10.3390/cells15171543 (PMC13564631; doi:10.3390/cells15171543)
Supplement: Supplementary file 1 [file cells-15-01543-s001.zip › cells-4469348-supplementary.pdf]

Supplementary Material

Supplementary Methods

Measurement of bone mass density (BMD)

For the evaluation of high-dose and long-term toxicity, ICR mice were used and treated orally with 100, 200, or 400 mg/kg of the compound for 28 days. Feed and water were supplied free to access. All animals fasted overnight, about 18 hours (with free access to water) before first administration and further for 3 hours after first administration, and sacrifice. Clinical signs were monitored at 24-hour intervals during the entire experimental period. At the end of the 28-day administration period, the mean bone mineral density (BMD) of the total body, left femur, and calvaria was measured individually via in vivo dual-energy X-ray absorptiometry (DEXA, InAlyzer, Korea). Animal procedures were performed in accordance with international guidelines for the care and use of laboratory animals and were approved by the Institutional Animal Care and Use Committee of Daegu Haany University (approval numbers DHU 2024-056).

Immunohistochemistry

Mouse Femur and calvaria were decalcified in decalcifying solution (24.4% formic acid, and 0.5N sodium hydroxide) for 3 days (changing the solution everyday), and then longitudinally trimmed. The bone structure, resorption, and mass were measured in the cortical or epiphyseal regions of the femur and the central zone of calvaria. Thickness of the cortical/plate bone were measured in the femurs' mid-shaft region and the central zone of the calvaria. Trabecular bone volume (TBV), and thickness of the femur cortical bone (Cbt) and the calvaria plate (Pbt) for structure and mass, and osteoclast cell number (Ocn) and osteoclast cell ratios on the bone surface OS/BS) on the left femur epiphyseal regions and central zone of calvaria were estimated for bone resorption.

Gene expression analysis

Gene expression data were obtained from the National Center for Biotechnology Information (NCBI) GEO database (http://www.ncbi.nlm.nih.gov/geo). The GSE252266 dataset was used to develop the gene signature. Raw expression data were first imported into BRB-ArrayTools for normalization and preprocessing according to the standard BRB-ArrayTools workflow. After normalization, genes were filtered based on an absolute log2 fold-change greater than 1, corresponding to at least a 2-fold difference in expression between groups. Differential expression analysis was then performed using the normalized data, and significantly altered genes were selected for downstream analysis. A volcano plot was generated using R version 4.3 to visualize the distribution of differentially expressed genes.

Measurement of antioxidant system

Prepared homogenates of heart samples were mixed with 0.1 ml of 25% trichloroacetic acid (Merck, USA), and then centrifuged at 4,200 rpm for 40 min at 4℃. GSH contents were spectrophotometerically measured at absorbance 412 nm using 2-nitrobenzoic acid (Sigma-Aldrich, USA). Decomposition of H_2O_2 in the presence of CAT was followed at 240 nm using spectrophotometer. CAT activity was defined as the amount of enzyme required to decompose 1 nM of H_2O_2 per minute, at 25℃ and pH 7.8. Results were expressed as U/mg protein. Measurements of SOD activities were made according to Sun et al. [1] SOD estimation was based on the generation of superoxide radicals produced by xanthine and xanthine oxidase, which react with nitrotetrazolium blue to form formazan dye. SOD activity was then spectrophotometerically measured at 560 nm by the degree of inhibition of this reaction and expressed as U/mg protein. One unit of SOD enzymatic activity is equal to the amount of enzyme that diminishes the initial absorbance of nitroblue tetrazolium by 50% during 1 min.

Supplementary Figures and Tables

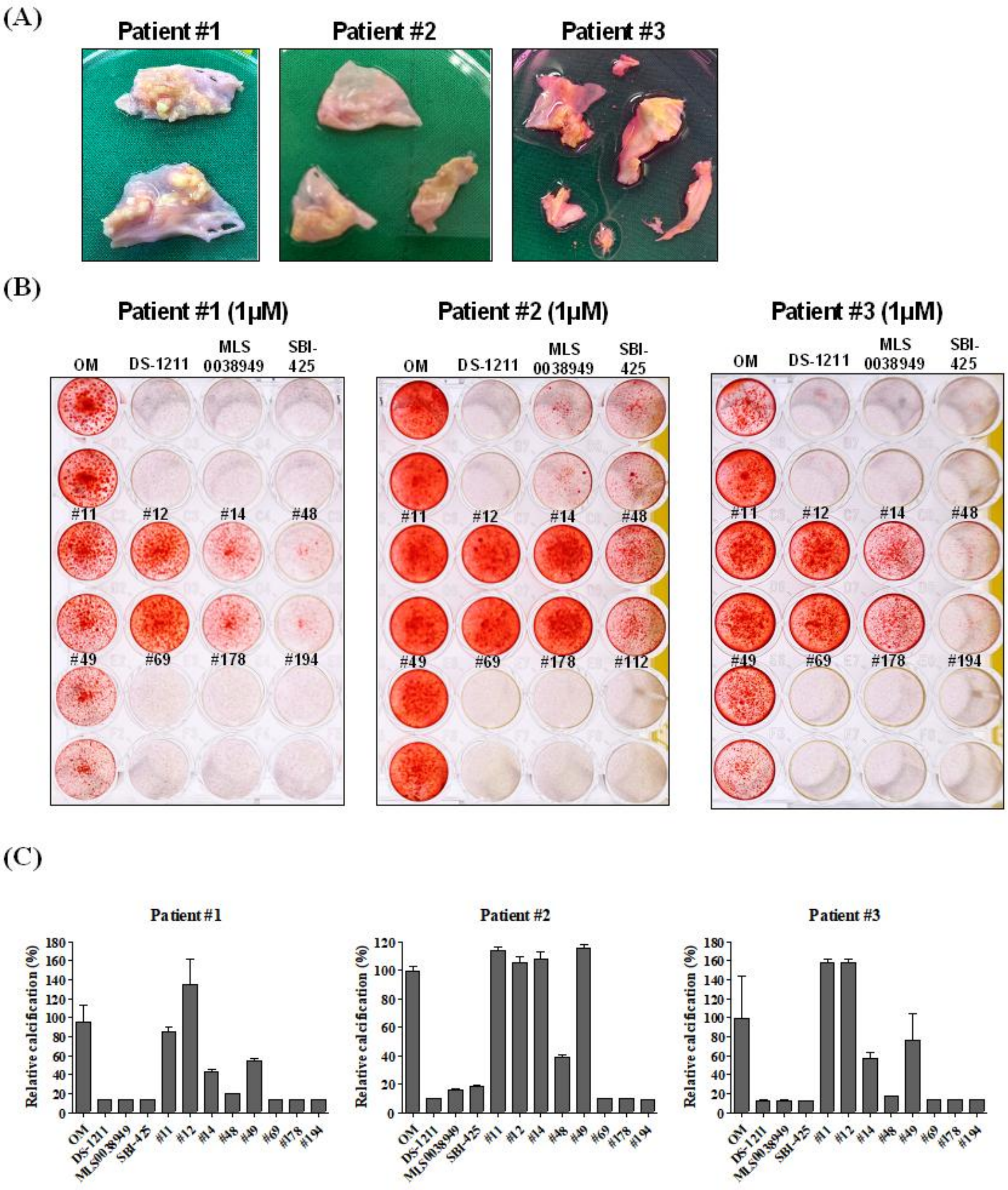

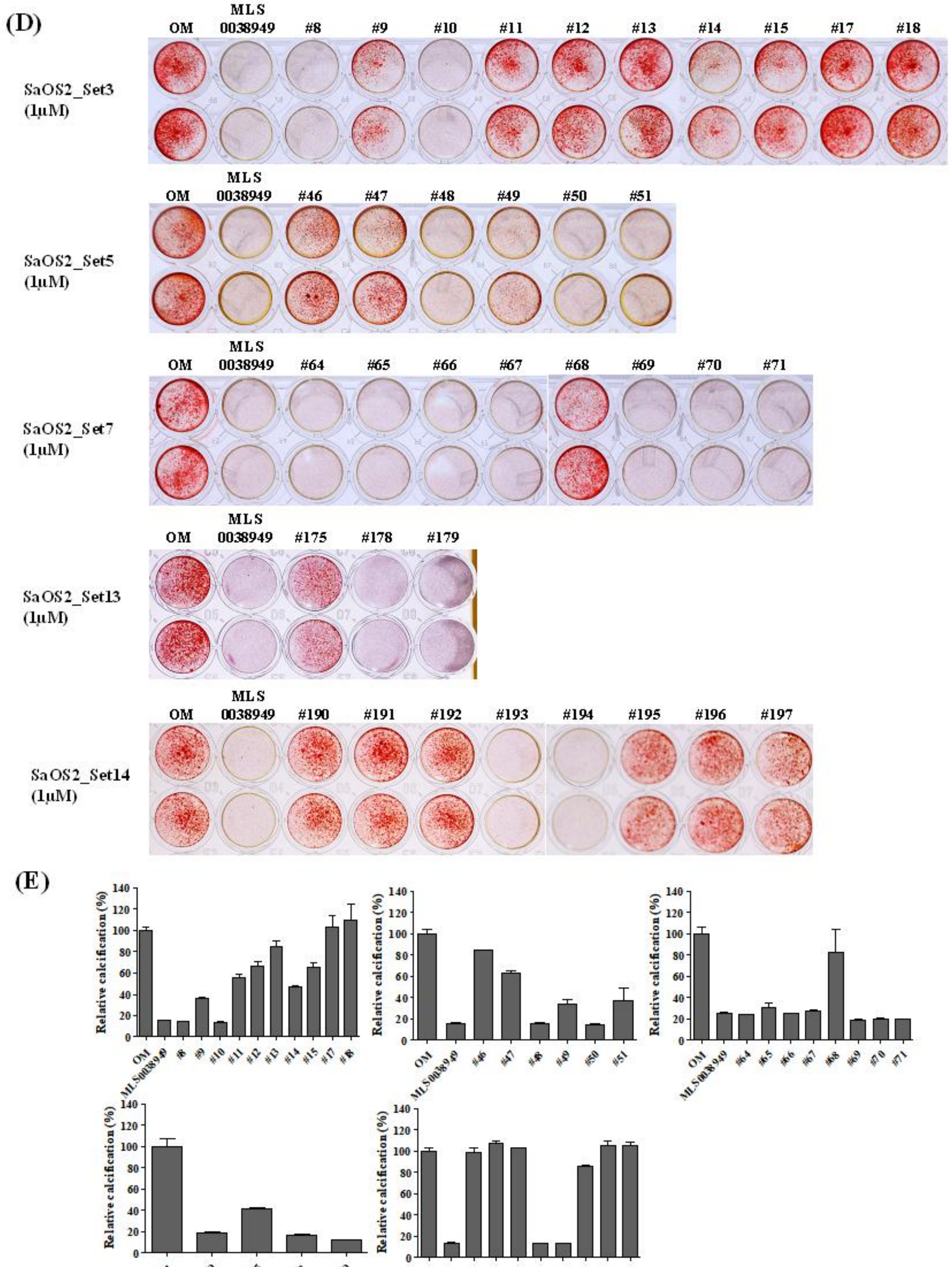

Supplementary Figure S1. Efficacy of novel TNAP inhibitors in VICs and SaOS-2 cells.((A) Gross morphology of aortic valve tissues obtained from CAVD patients. (B) Alizarin Red S staining of candidate TNAP inhibitors in patient-derived VICs. (C) Quantitative analysis of relative calcification levels in patient-derived VICs. (D) Alizarin Red S staining of candidate TNAP inhibitors in SaOS-2 cells. (E) Quantitative measurement of relative calcification levels in SaOS-2 cells.

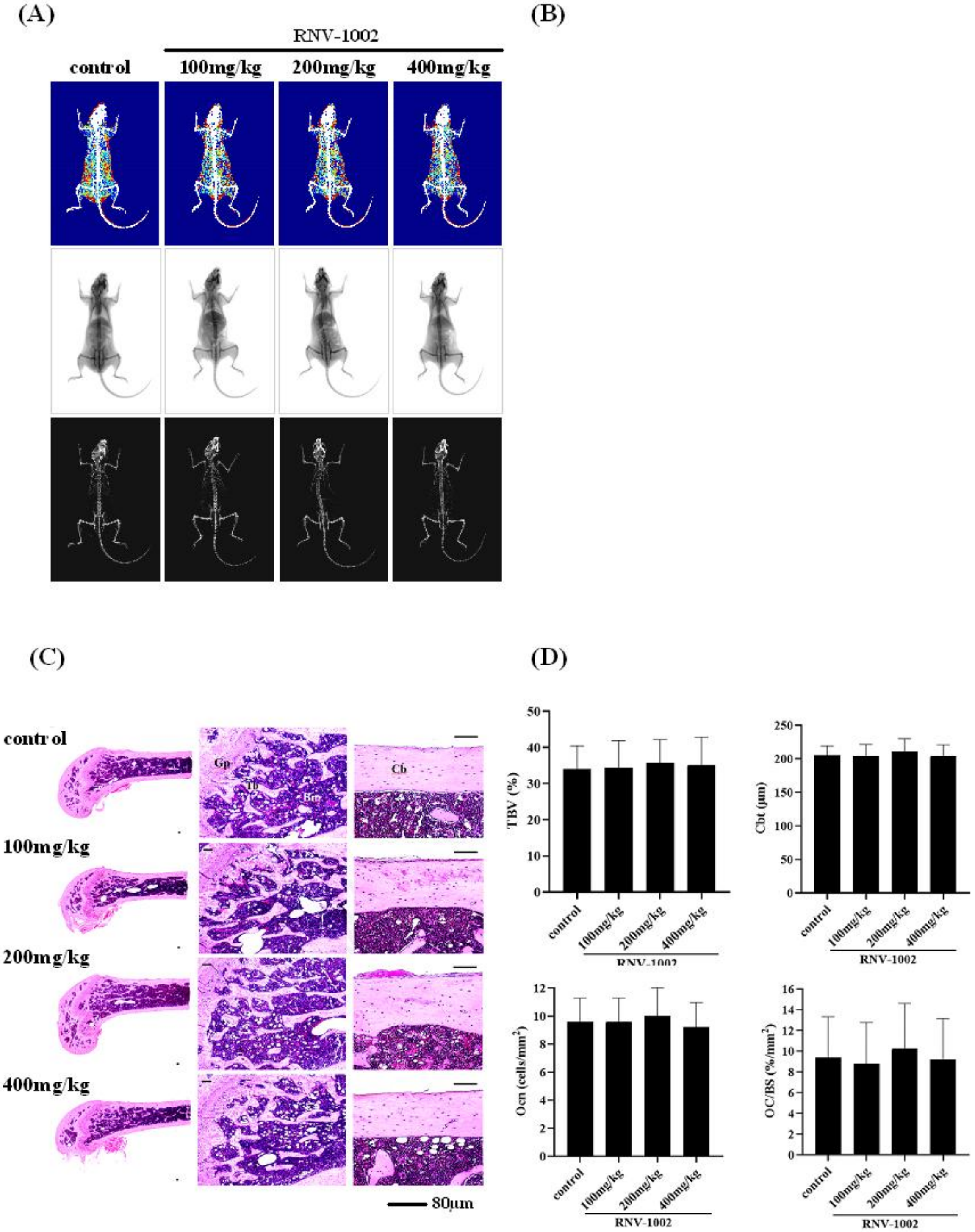

Supplementary Figure S2. Effects of RNV-1002 on bone. (A) DEXA images of whole-body scans from control, RNV-1002–treated mouse (100, 200, and 400 mg/kg). (B) Quantitative analysis of BMD in total body, left femur, and calvaria from control, RNV-1002–treated mouse. (C) H&E staining in femur from control, RNV-1002–treated mouse (Scale bar = 80 μm). (D) Quantitative analysis of bone parameters, including TBV, Cbt, Ocn, OC/BS from control, RNV-1002–treated mouse. Data are shown as mean ± SD. * p-value < 0.05.

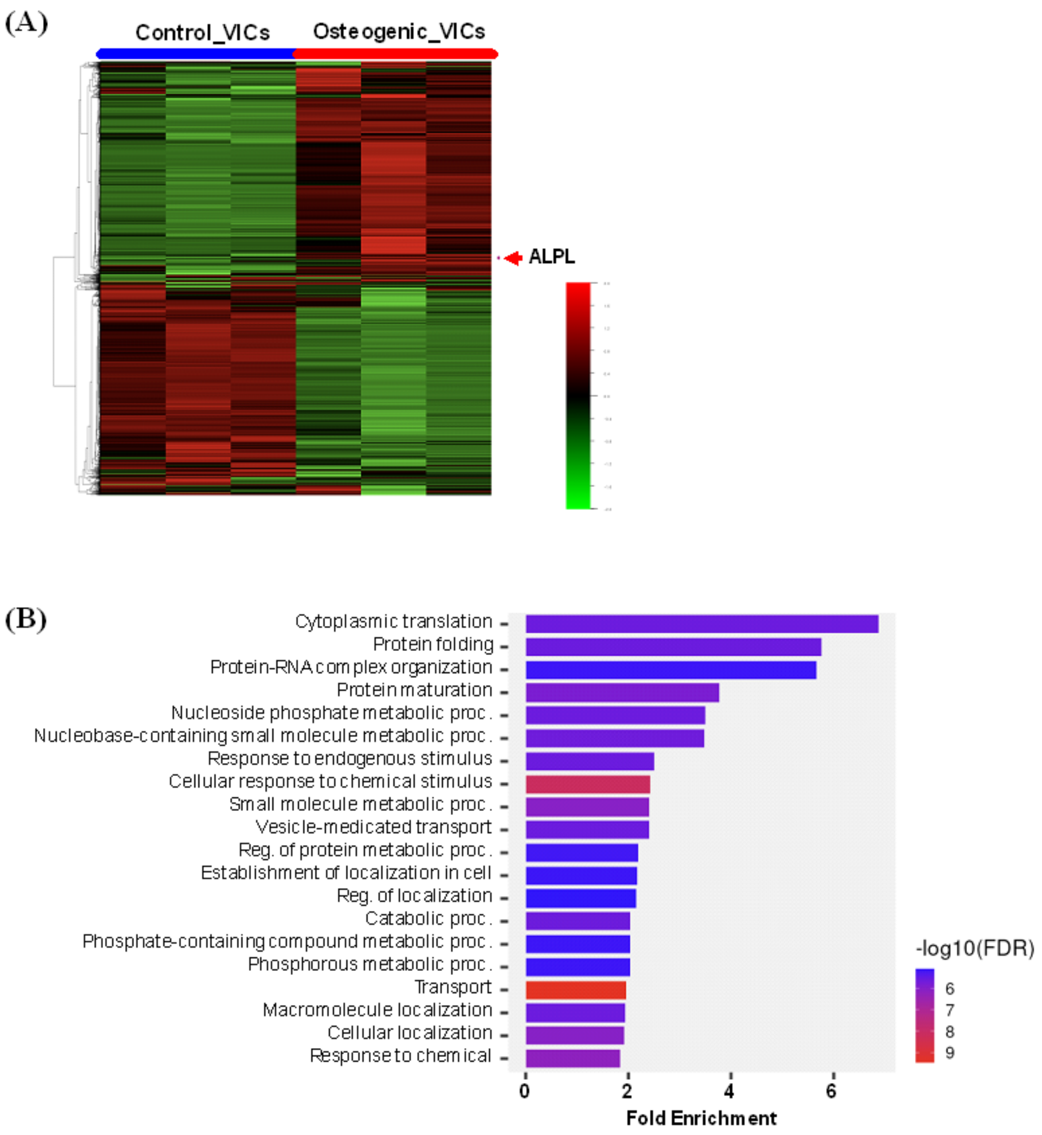

Supplementary Figure S3. NGS analysis of VICs with/without CAVD. ((A) Heatmap of **ALPL** expression in osteogenic valvular interstitial cells (VICs) analyzed using NGS data (GSE252266). (B) Gene Ontology annotation of 277 genes identified from gene expression analysis.

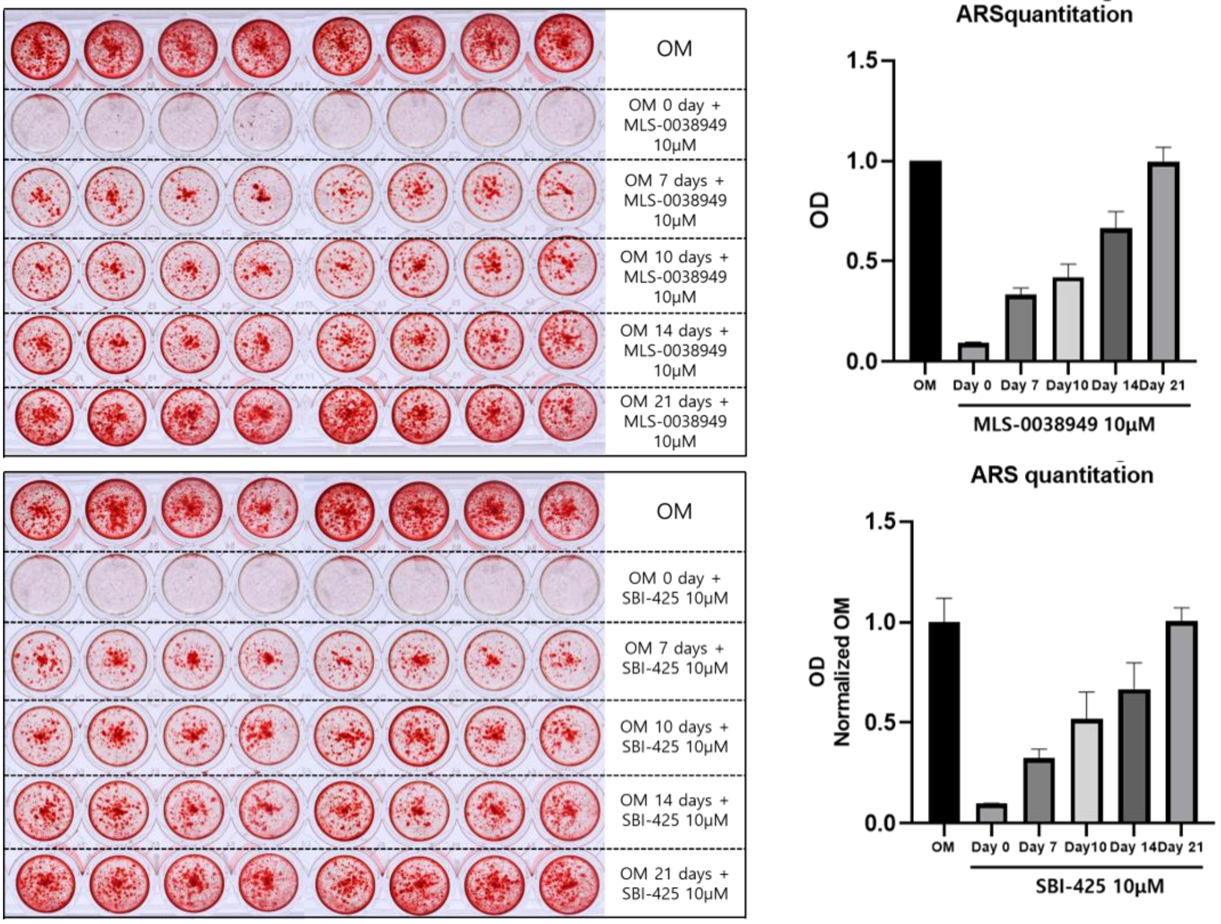

Supplementary Figure S4. Delayed TNAP inhibition attenuates ongoing calcification in primary human valve interstitial cells (hVICs). Human VICs were cultured in osteogenic medium (OM) for 28 days to induce calcification. TNAP inhibitors were added at indicated post-induction time points (Day 0, 7, 10, 14, and 21) to evaluate the therapeutic potential on ongoing osteogenesis. (A) Representative Alizarin Red S (ARS) staining images showing the effect of delayed treatment with MLS-0038949 (10μM). (B) Spectrophotometric quantification of ARS staining shown in (A). (C) Representative ARS staining images showing the effect of delayed treatment with SBI-425 (10μM). (D) Spectrophotometric quantification of ARS staining shown in (C). Data are presented as mean ± SD (n=8 biological replicates).

Supplementary Table S1. IC_{50} of TNAP inhibitors

Comp. No.	IC_{50} (nM)	Comp. No.	IC_{50} (nM)
DS-1211	9.6	#64	216
MLS0038949	219.5	#65	459
SBI-425	16.0	#67	579.2
#8	225.6	#68	>10μM
#9	72.0	#69	29.9
#10	177.2	#70	22.1
#11	305	#71	35.1
#12	2,010	#175	1,730
#13	3,520	#178	6.5
#14	137.7	#179	9.2
#15	1,197	#190	7,136
#17	>10μM	#191	>10μM
#18	>10μM	#192	9,777
#46	2,061	#193	14.9
#47	6,551	#194	5.6
#48	271.4	#195	3,964
#49	1,075	#196	3,628
#50	240	#197	>10μM
#51	369.8		

Supplementary Table S2. The protein expression list by proteomic analysis.

Up-regulated	Down-regulated						
YWHAH	XPNPEP1	ASPH	HSP90AA1	MYO1E	GALNT2	CPNE1	HSP90B1
CMA1	PACSIN2	SFXN1	SPTAN1	EIF4A3	RABGGTA	G6PD	RPS12
IGLV9-49	HNRNPA3	ACTR1A	ATP1B3	BDH2	EPRS1	NCALD	CORO1B
ALPL	SGCE	FBL	APRT	ACOT9	DNAJB11	SART3	TMEM87A
A2M	CALHM5	MYO1B	ACADM	BLVRA	CTSB	LPL	C11orf68
C4A	EIF4E	ERP44	RPLP0	NUCB2	SMAD4	PRAF2	CHI3L1
MAOA	PPP3CA	SEMA3B	ESYT2	S100A9	LEFTY1	SURF4	CAPN2
MX2	AP2B1	TAPBP	RAD23A	DNPEP	COPG1	GNB1	TMPO
ANGPTL8	RPL27	ANP32A	ZC3HAV1	CPPED1	ADK	PRKDC	CCT5
PTTG1IP	PPP2R1A	CYB5R3	RO60	QNG1	ATP1B1	DYNC1LI2	PURA
KNG1	RCC2	CMAS	LTBP1	RPL7A	NDUFS7	RAC1	TK2
IGHG1	DPYSL3	MACF1	IMPDH2	HSP90AB1	LTA4H	HNRNPA2B1	VPS35L
SERPIND1	ERGIC1	PDGFRB	SLC25A11	NUBP2	FERMT3	PLSCR4	UCHL1
IGHV3-7	SERPINB9	NPEPPS	STT3B	NID2	PPIB	TWF1	PDCD6IP
AGT	RPSA	CAVIN1	PGRMC2	RASGRP2	NDUFV1	ENO1	LCN2
APOA4	MYO1D	MRE11	LTBP4	ENTPD6	OSBPL8	PDCD4	SPP2
AKAP9	LASP1	CAND1	MVP	KLC1	TPI1	CHST14	NAPA
ITIH4	ENG	GOT2	SNX33	MOB3C	ACTN4	RPL9	EHD1
MXRA8	IAH1	KIF5B	RPL32	GBF1	PABPC4	PEBP1	CUL3
HLA-DRB1	CAMK2D	RAB6A	PLCXD3	THEM6	CLIC1	CIRBP	DHX9
PON1	PDCD6	LTBP2	ARL2	NDRG1	RPS3A	EIF3B	HSPA1B
C3	NEK7	MESD	SCRN1	ANXA5	TNS1	OAT	HMGB1
SPARCL1	PCBP2	HNRNPC	DOCK7	UBA5	COPA	SULF1	PPP5C
PTGDS	CDK4	EIF3A	EIF3D	PRDX4	LRP1	GP1BA	BCLAF1
IGKV1D-39	PDHA1	PRDX1	SMARCA4	PLPP3	GSPT1	ANXA4	TCP1
IGHV3-64D	FKBP7	NCL	CUL2	LDHA	TRIP10	ANTXR2	PMM2
EIF4G3	RBP1	ABHD14B	SF3B2	PKM	FLNA	FASN	CARS1
SAA4	PCBD1	COX5B	SSRP1	DOK2	C11orf54	NDUFS1	BTK
PSMD3	SELENBP1	ANKFY1	GPX1	RASA3	NID1	DES	PDCD10
APOC1	RANBP2	FBLN2	GNPDA1	KRT5	RAP1B	HAPLN1	ALDOA
ALB	ILF2	EIF3L	MAP1B	PRELP	PPIA	NECAP2	TMX1
AHSG	CAVIN2	CPZ	DDX5	MYLK	DEK	PPIL1	ATP6V0D1
SERPINA1	COPB1	EIF5	PFKL	SH3PXD2B	HNRNPR	PRKCB	COX6B1
APOB	PMVK	AGO1	NANS	MYH9	NUDT5	H1-10	
	ESYT1	DDB1	PLS3	ARMCX1	DDX17	ACO2	

References

1. Sun, Y.; Oberley, L.W.; Li, Y. A Simple Method for Clinical Assay of Superoxide Dismutase. *Clin. Chem.* **1988**, *34*, 497–500, doi:10.1093/CLINCHEM/34.3.497.
